# Assessing trait contribution and mapping novel QTL for salinity tolerance using the Bangladeshi rice landrace Capsule

**DOI:** 10.1186/s12284-019-0319-5

**Published:** 2019-08-13

**Authors:** M. Akhlasur Rahman, Michael J. Thomson, Marjorie De Ocampo, James A. Egdane, M. A. Salam, M. Shah-E-Alam, Abdelbagi M. Ismail

**Affiliations:** 1 International Rice Research Institute, DAPO Box 7777, Metro Manila, Philippines; 2 Bangladesh Rice Research Institute, Gazipur, 1701 Bangladesh; 3 Department of Soil and Crop Sciences, Texas A&M University, College Station, TX 77843 USA; 4 Bangladesh Agricultural University, Mymensingh, 2202 Bangladesh

**Keywords:** Novel QTL, Path analysis, Salinity tolerance mechanisms, Salt tolerant *indica* landrace, Simple sequence repeats

## Abstract

**Background:**

Salinity is one of the most widespread abiotic stresses affecting rice productivity worldwide. The purpose of this study was to establish the relative importance of different traits associated with salinity tolerance in rice and to identify new quantitative trait loci (QTL) conferring tolerance to salinity at seedling stage. A total of 231 F_2:3_ plants derived from a cross between a sensitive variety BRRI dhan29 (BR29 hereafter) and Capsule, a salt tolerant Bangladeshi *indica* landrace, were evaluated under salt stress in a phytotron.

**Results:**

Out of the 231 F_2_ plants, 47 highly tolerant and 47 most sensitive lines were selected, representing the two extreme tails of the phenotypic distribution. These 94 plants were genotyped for 105 simple sequence repeat (SSR) and insertion/deletion (InDel) markers. A genetic linkage map spanning approximately 1442.9 cM of the 12 linkage groups with an average marker distance of 13.7 cM was constructed. QTL were identified on the long arm of chromosome 1 for Na^+^ concentration, K^+^ concentration, Na^+^-K^+^ ratio and survival; chromosome 3 for Na^+^ concentration, survival and overall phenotypic evaluation using the Standard Evaluation system (SES); and chromosome 5 for SES. A total of 6 pairwise epistatic interactions were also detected between QTL-linked and QTL-unlinked regions. Graphical genotyping indicated an association between the phenotypes of the extreme families and their QTL genotypes. Path coefficient analysis revealed that Na^+^ concentration, survival, Na^+^-K^+^ ratio and the overall phenotypic performance (SES score) are the major traits associated with salinity tolerance of Capsule.

**Conclusions:**

Capsule provides an alternative source of salinity tolerance aside from Pokkali and Nona Bokra, the two Indian salt tolerant landraces traditionally used for breeding salt tolerant rice varieties. Pyramiding the new QTL identified in this study with previously discovered loci, such as *Saltol*, will facilitate breeding varieties that are highly tolerant of salt stress.

**Electronic supplementary material:**

The online version of this article (10.1186/s12284-019-0319-5) contains supplementary material, which is available to authorized users.

## Background

Agriculture in unfavorable coastal rice production environments faces enormous threats due to climate change, challenging future food security and contribution to achievement of sustainable development goals (Jagadish et al. 2012). Abiotic stresses have become increasingly important because more than 50% of crop yields are negatively affected by these stresses (Vij and Tyagi 2007), with salinity causing major losses (Chinnusamy et al. 2005). Rice (*Oryza sativa* L.), a staple food for half of the world’s population, is sensitive to salinity. In coastal areas, salinity tolerance at seedling stage is crucial for crop establishment because soil salinity is usually high at the beginning of the rainy season; however, tolerance is a complex quantitative trait. The dissection of the genetic basis of tolerance to abiotic stresses like salinity has greatly improved following the introduction of molecular platforms that enable the identification of quantitative trait loci (QTL) governing relevant genetic variation in crops (Tanksley 1993; Ribaut and Hoisington 1998; Flowers et al. 2000; Koyama et al. 2001; Munns 2005; Tuberosa and Salvi 2006; Thomson et al. 2010; Hossain et al. 2015; Ismail and Horie 2017; Rahman et al. 2017). The identification of a QTL is one step in determining the genes associated with variation in a polygenic trait such as salinity tolerance. Moreover, direct selection of superior genotypes based on salinity tolerance phenotype is not as effective as selecting for the underlying physiological and agronomic components based on QTL underlining particular traits.

The association of plant characters and tolerance assumes special importance in the formulation of selection criteria for tolerance to salinity. When establishing the relationship between stress and genotypic tolerance, correlation analysis is a useful tool. It provides an indication of the degree of association between two variables, which are considered to be interdependent (Gomez and Gomez 1984). Genetic correlation analysis simply measures the association between two traits, but cannot elucidate the related mechanisms among them. Path analysis can dissect the correlation coefficients into direct and indirect effects, and quantify the relative contribution of each component to the overall correlation (Kang 1994; Condon et al. 2004). It can be used to quantify a perceived biological relationship through partitioning of correlation coefficients (Sokal and Rohlf 1995; Gravois and Helms 1992). If an indirect relationship exists between two variables, path analysis can help elucidate to what extent other variables are involved or affecting the relationship. Using path analysis, the variables contributing the most tolerance to salinity can be identified.

Exploring rice germplasm with useful traits is a key step for pre-breeding programs to identify useful alleles. Capsule (locally known as ‘Capsail’), a salt tolerant, widely adapted Bangladeshi *indica* landrace, has been identified as a good donor for salinity tolerance having superior phenotype for Na^+^ exclusion and early seedling vigor (Rahman et al. 2016). We anticipated that Capsule would have novel genetic variation for salinity tolerance, in comparison with Pokkali and Nona Bokra, which have been well exploited by physiologists and molecular biologists as salt-tolerant genotypes. The superior tolerance of salinity in Pokkali is attributed to two main traits: its capacity to maintain a low Na^+^-K^+^ ratio in the shoot tissue and its faster growth rate under saline conditions, which helps in diluting the salt and reducing toxicity stress within the tissue (Walia et al. 2005; Ismail et al. 2007; Thomson et al. 2010). Mapping of QTL is important to augment our knowledge of the inheritance and genetic architecture of quantitative traits (Mackay 2001). It also facilitates developing markers for complex traits and combining favorable alleles in breeding lines. The present study aims to (i) establish the relative importance of different traits associated with salinity tolerance and to delineate their importance as individual traits or when combined, ii) identify novel QTL conferring salinity tolerance at seedling stage from the tolerant landrace Capsule using selective genotyping.

## Methods

### Choice of parents

The two parents used to develop the mapping population are Capsule and BRRI dhan29. Capsule is an *indica* landrace tolerant of salt stress at seedling stage and grown in southern coastal region of Bangladesh that are affected by salinity. It is tall (150–155 cm), moderately photoperiod sensitive, produces vigorous seedlings, has long, broad, and droopy leaves, about 6–8 tillers, and with panicle length of 20–25 cm. Grains are typically awnless; bold and blackish in color, with poor grain quality. Its yield is low (1.5–2.0 t ha^− 1^) and mature in 110–115 days (Rahman et al. 2016). BRRI dhan29 (BR29) is a high yielding popular *indica* rice variety with: i) medium stature; 95–105 cm, awnless, with medium slender grains; produce 15–20 tillers and yield 6.0–6.5 t ha^− 1^. It is photoperiod insensitive, but sensitive to salt stress at seedling stage.

An F_2_ population and its F_2:3_ families were developed and used in this study for mapping traits associated with salinity tolerance.

### Phenotypic evaluation of the mapping population

#### Growing condition

This experiment was carried out during the dry season at the International Rice Research Institute (IRRI), Los Baños, Philippines. Seedlings were phenotyped in a hydroponic system in a phytotron set at 29 °C/21 °C day/night temperature and 70% relative humidity. Seeds were heat treated for 5 d in a convection oven set at 50 °C to break their dormancy, surface-sterilized with a fungicide (Vitavax-200, Syngenta) and rinsed with distilled water, then placed in petri dishes lined with moistened filter paper and incubated at 30 °C for 48 h to germinate. Two pre-germinated seeds were sown per hole on styrofoam seedling floats suspended on distilled water in 10 L plastic trays for 3 d, after which a culture solution (Yoshida et al. 1976) was used. At 14 d after seeding, salt stress was introduced by adding NaCl to the culture solution until an electrical conductivity of 12 dS m^− 1^ was reached. Silicon in the form of sodium metasilicate 9 hydrate (4.5 mg L^− 1^) was added to avoid lodging. The nutrient solution was acidified daily to pH 5.0 to avoid Fe-deficiency, and renewed every 7 d.

#### Agronomic characterization

A total of 231 F_2:3_ individuals were characterized for selected agronomic and physiological traits. Phenotypic evaluation based on SES scores (Standard Evaluation System; IRRI 2014) was used to assess the symptoms of salt stress injury of seedlings, with a score of 1 indicating highly tolerant and 9 as highly sensitive or dying plants. Individual plants were harvested 25 d after salinization, dried in an oven set at 70 °C for 3 d and then weighed. The fully expanded third leaf was separately harvested and dried at 70 °C for 3 d and weighed, then analyzed for sodium and potassium concentrations (Flowers and Yeo 1981; Moradi and Ismail 2007; Rahman et al. 2016). Surviving plants were counted 3 weeks after salinization and survival percentage was calculated based on number of seedlings at the start of the treatment. Shoot length was measured from the base of the stem to the tip of the tallest leaf.

#### Physiological characterization

The fully expanded 3rd leaf collected from each F_2:3_ family was washed 3 times with deionized water before drying. Two subsamples from each replication were weighed and placed in a test tube and 10 ml of 100 mM acetic acid was added to each tube. The samples were then digested for 2 h in a water bath set at 90 °C. After digestion, 1 ml of the liquid phase was transferred to a test tube and diluted by adding 9 ml of 100 mM Acetic acid and read in an Atomic Absorption Spectrophotometer (PerkinElmer*,* AAnalyst 200, FL, USA) to determine sodium and potassium concentrations.

#### SPAD readings

Chlorophyll concentration in leaves was estimated non-destructively before harvesting, using a handheld SPAD meter (Minolta 502, Japan). Five fully expanded leaves were selected for each entry in each replication and the average of 30 readings (six readings on each leaf) was averaged for each entry.

### Correlation analyses for trait associations

A total of 94 individuals (extremes of 47 tolerant and 47 sensitive) families were selected from the 231 F_2:3_ families based on phenotypic traits (SES, survival, Na^+^ concentration) and used for further analysis. Correlation coefficients for the associations between different traits were estimated using QGene 4.0 software (Joehanes and Nelson 2008). The statistical significance of the correlations was calculated according to Fisher and Yates (1938).

### Path analysis

Path analysis was used to partition the relative contributions of salt stress tolerance components using standardized partial-regression coefficients. The correlation coefficients can be separated into the direct and indirect influences that one variable has on another (Wright 1922; Dewey and Lu 1959). This technique involves partitioning of the correlation coefficients to determine direct (unidirectional pathways, P) and indirect influence through alternate pathways (pathway P x correlation coefficient r) of various variables over salt stress tolerance. Salinity tolerance was considered the resultant variable and the others as causal variables:

$$ {\displaystyle \begin{array}{l}{\mathrm{r}}_{1\mathrm{y}}={\mathrm{P}}_{1\mathrm{y}}+{\mathrm{r}}_{12}{\mathrm{P}}_{2\mathrm{y}}+{\mathrm{r}}_{13}{\mathrm{P}}_{3\mathrm{y}}+{\mathrm{r}}_{14}{\mathrm{P}}_{4\mathrm{y}}\left[{\mathrm{x}}_1\right]\\ {}{\mathrm{r}}_{2\mathrm{y}}={\mathrm{r}}_{12}{\mathrm{P}}_{1\mathrm{y}}+{\mathrm{P}}_{2\mathrm{y}}+{\mathrm{r}}_{23}{\mathrm{P}}_{3\mathrm{y}}+{\mathrm{r}}_{24}{\mathrm{P}}_{4\mathrm{y}}\left[{\mathrm{x}}_2\right]\\ {}{\mathrm{r}}_{3\mathrm{y}}={\mathrm{r}}_{13}{\mathrm{P}}_{1\mathrm{y}}+{\mathrm{r}}_{23}{\mathrm{P}}_{2\mathrm{y}}+{\mathrm{P}}_{3\mathrm{y}}+{\mathrm{r}}_{34}{\mathrm{P}}_{4\mathrm{y}}\left[{\mathrm{x}}_3\right]\\ {}{\mathrm{r}}_{4\mathrm{y}}={\mathrm{r}}_{14}{\mathrm{P}}_{1\mathrm{y}}+{\mathrm{r}}_{24}{\mathrm{P}}_{2\mathrm{y}}+{\mathrm{r}}_{34}{\mathrm{P}}_{3\mathrm{y}}+{\mathrm{P}}_{4\mathrm{y}}\left[{\mathrm{x}}_4\right]\end{array}} $$

Where:

Y = overall phenotypic performance for salt tolerance

Pij represents path coefficients, where i indicates the effect and j the cause

rij represents linear correlation coefficients.

The hypothesized equations were used to determine the causal relationships between the response variable, overall phenotypic performance i.e. SES and the four component variables, Na^+^ concentration (x_1_), K^+^ concentration (x_2_), % Survival (x_3_), and Na-K ratio (x_4_) etc. Path coefficients are represented by P_15_, P_25_, P_35_ and P_45_, which correspond to direct effects on overall performance under salt stress, of Na^+^ concentration, K^+^ concentration, % Survival and Na-K ratio. For any two-component variables (e.g. x_1_ and x_2_), their correlation (r_12_) multiplied by the path coefficient of the second component variable (e.g. P_25_) estimates the indirect effect of one component (x_1_) on x_5_ through the effect of another component (x_2_). The residual is the remaining portion of overall phenotypic performance (salinity tolerance) not accounted for by the studied parameters.

$$ \mathrm{Residual}\ \mathrm{effect}\ \left(\mathrm{R}\right)=\sqrt{1.0-\left(\mathrm{P}14\mathrm{r}14+\mathrm{P}24\mathrm{r}24+\mathrm{P}34\mathrm{r}34\right)} $$

### Identification of markers polymorphic between the two parents

A set of 450 microsatellite and insertion/deletion (InDel) markers spanning all 12 chromosomes was screened between Capsule and BR29 parents, and 105 markers that detected polymorphism between the two parents were used for genotypic analysis of the population. These markers were identified from SSR/InDel primers available in previously published rice genetic and sequence maps (Temnykh et al. 2001; McCouch et al. 2002; Shen et al. 2004; IRGSP (International Rice Genome Sequencing Project) and Matsumoto 2005) and the public domain (http://www.gramene.org). Segregation distortion (SD) in the F_2_ generation was investigated with a chi-square (χ2)-test for goodness-of-fit to the segregation ratios expected (1:2:1) under Mendelian inheritance and applying a significance level of *P* < 0.05. The probability value was *P >* 0.05 for non-distorted markers.

### Genotyping of the population and construction of a genetic linkage map

Genomic DNA of 231 individuals of an F_2_ population from the Capsule/BR29 cross was extracted from young leaves of 3-week old plants using a standard DNA miniprep method. Marker genotype data was obtained by running SSR markers using 15 μL PCR reactions on 96-well plates with a 55 °C annealing temperature (MJ Research and G-Storm thermal cyclers), and then run on 6% acrylamide gels at 100v (Dual Triple-Wide Mini-Vertical System, C.B.S. Scientific, CA, USA) followed by SYBR-Safe staining (Invitrogen), gel documentation (Alpha Innotech), and manual scoring. The molecular map of the 105 markers was based on the order of their position on the physical map based on the Nipponbare genomic sequence (www.gramene.org), and the marker distances were calculated by multiplying the Mb positions by a factor of 4 to obtain an equivalent estimate of cM.

### QTL analysis

The 94 selected F_2_ plants were used for marker analyses and QTL identification representing the two extreme tails of the response distribution. This proportion of selection corresponds to a selection intensity *i* = 1.14 (Falconer 1989). These progenies were genotyped for 105 SSR and InDel markers separated by an average distance of 11.6 cM. Three analytical approaches were used to identify QTL for salinity tolerance and to estimate their phenotypic effects. First, QTL analysis was performed using QGene 4.0 software (Joehanes and Nelson 2008; www.qgene.org), to determine the association between individual marker loci and salt tolerance related traits.To be more precise about the location of the identified QTL for salt tolerance, interval mapping (IM) and composite interval mapping (CIM) analysis were conducted. The minimal LOD value required to declare a QTL as significant was obtained empirically from 1000 permutation tests (Churchill and Doerge 1994). The proportion of the total phenotypic variation explained by each QTL was calculated as *R*^*2*^ value (*R*^*2*^ = PVE, phenotypic variation explained by the QTL). Forward cofactor selection method commands were used for the CIM. The proportion of phenotypic variance (*R*^*2*^ value) and additive effects were determined for each trait.

The second approach to identify marker-linked QTL was the analysis of distributional extremes. In this method, the 47 salt tolerant families (20.35% of the total) and the 47 salt sensitive families selected as the extreme tails of the F_2:3_ population were genotyped and the marker-allele frequencies within each class were calculated at all of the 105 marker loci. For unidirectional selective genotyping, the variance of allele frequency for each marker was calculated as a binomial variance (*s*^*2*^*q = pq/*2 *N*), where *p* and *q* are the corresponding allele frequencies at a given marker locus and *N* is the number of individuals genotyped at that locus (Falconer 1989). Marker allele frequency differences *(p*_*r*_*-p*_*s*_*)* between the selected salt-tolerant and salt-sensitive progeny families were estimated for each marker locus, where *p*_*r*_ is the frequency of the *i*th allele at the *k*th marker locus among the salt-tolerant families and *p*_s_ is the frequency of the *i*th allele at the *k*th marker locus among the salt-sensitive families. A trait-based marker analysis (TBA; also known as selective genotyping), which measures differences in marker-allele frequencies between selected classes, was used to identify marker linked QTL (Lebowitz et al. 1987; Lander and Botstein 1989; Darvasi and Soller 1992; Foolad et al. 1997). Allele frequency differences were found to be significant when (*p*_*r*_*-p*_*s*_) *≥* 2σ_*p*_, where σ_*p*_ *=* (*p*_*r*_*q*_*r*_*/*2*N*_*r+*_*p*_*s*_*q*_*s*_*/*2*N*_*s*_)^*½*^ is the standard error of the difference between marker-allele frequencies, *Nr* is the number of salt-tolerant families, and *Ns* is the number of salt-sensitive families (Falconer 1989; Darvasi and Soller 1992; Foolad et al. 1997). This test provides a confidence of greater than 95% on the identified QTL (Steel and Torrie 1980; Lebowitz et al. 1987; Foolad et al. 1997). For each marker at which a significant effect was detected by trait-based analysis of data from bidirectional selective genotyping, the proportion of phenotypic variance explained (*R*^*2*^_p_) was estimated as:

$$ {R^2}_{\mathrm{p}}={d^2}_{Ps}/{i}^2\ \left[ Ps\left( 1- Ps\right)\right] $$

Where *d*_*Ps*_ is the difference in allele frequencies between the upper and lower selected tails and *i* is standardized selection differential (Falconer 1989).

The third approach is detection of pair-wise epistatic interactions among QTL-linked markers (QTL vs QTL), between QTL-linked and QTL-unlinked markers (QTL vs background) and among QTL-unlinked markers (between complementary loci). These digenic interactions between marker loci were determined using MapManager QTX (Manly et al. 2001). The QTL nomenclature followed the system proposed by McCouch, and CGSNL (Committee on Gene Symbolization, Nomenclature and Linkage, Rice Genetics Cooperative) (2008). The graphical genotype map (GGT) can visually present the contribution of each parental genome in the selected segregants and thus facilitate selection and evaluation of desirable individuals in the mapping population. This was also used to confirm the QTL effect. The GGT map was constructed only for those chromosomes where major QTL were identified for salt tolerance during seedling stage and the individuals that provide the most linkage information are those whose genotype can be most clearly inferred from the phenotype, i.e., the progenies that deviate most from the phenotypic mean (Ayoub and Mather 2002), the GGT maps were depicted for the extremes.

## Results

### Responses of the parental lines and the selected F_2:3_ progeny to salt stress

**Fig. 1:**
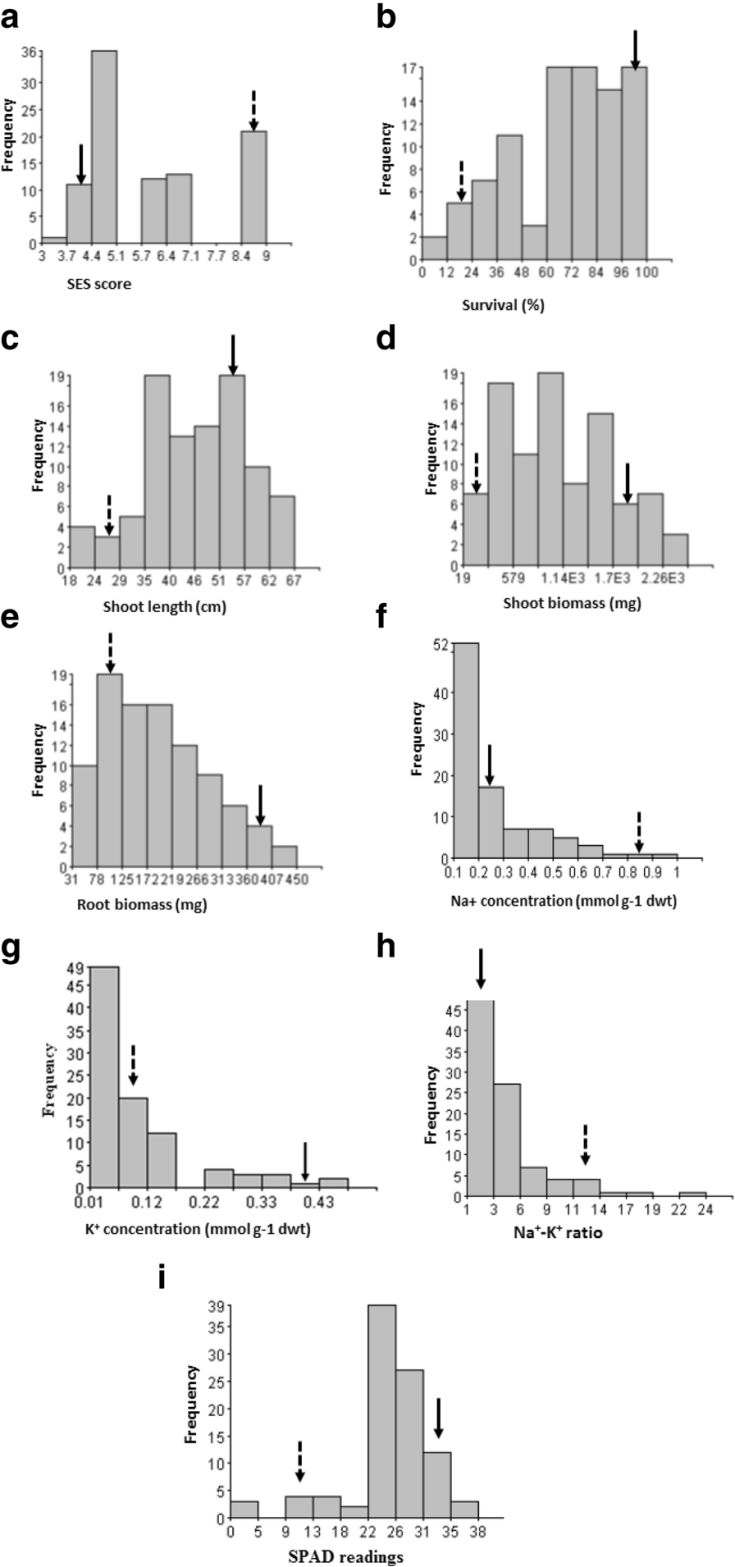
Frequency distribution (number of plants) of the F_2:3_ individuals for traits associated with salinity tolerance at seedling stage; **a** SES score, **b** survival **c** shoot length **d** shoot biomass **e** root biomass **f** Na^+^ concentration **g** K^+^ concentration **h** Na^+^-K^+^ ratio, and **i** SPAD readings. Solid and dotted arrows indicate the phenotypic value of the tolerant parent Capsule and sensitive BR29, respectively

A total of 231 F_2:3_ lines derived from a cross between a sensitive variety BRRI dhan29 (BR29 hereafter) and Capsule, a salt tolerant Bangladeshi *indica* landrace, were evaluated under salt stress in a phytotron. The genotypes in the F_2_ population differed significantly (*P* < 0.05) for all traits (Table 1, Fig. 1), indicating the presence of sufficient genetic variability.

**Table 1:** Correlation coefficients for the associations among Na^+^ concentration, overall phenotypic score (SES), survival, K^+^ concentration, Na^+^-K^+^ ratio, shoot and root biomass, and shoot length in F_2:3_ population of a cross between Capsule (salt tolerant) and BR29 (salt sensitive) subjected to salt stress at seedling stage

Trait	Na^+^ conc.	SES	Survival (%)	K^+^ conc.	Na^+^-K^+^ ratio	Biomass (mg)		Shoot length (cm)
						Shoot	Root	
SES	0.692^**^							
Survival	−0.709^**^	−0.795^**^						
K^+^ concentration	−0.299^**^	− 0.184	0.363^**^					
Na^+^-K^+^ ratio	0.476^**^	0.440^**^	−0.422^**^	−0.280^**^				
Biomass (shoot)	−0.632^**^	−0.815^**^	0.692^**^	0.262^*^	−0.480^**^			
Biomass (root)	−0.615^**^	−0.809^**^	0.723^**^	0.298^**^	−0.483^**^	0.930^**^		
Shoot length (cm)	−0.667^**^	−0.739^**^	0.774^**^	0.278^**^	−0.417^**^	0.747^**^	0.721^**^	
SPAD value	−0.617^**^	−0.541^**^	0.504^**^	0.041	−0.380^**^	0.462^**^	0.436^**^	0.517^**^

*conc.* concentration

^*^, ^**^ significant at *P* < 0.05 and *P* < 0.01, respectively; Tabulated t-value at 5% level = 1.989, at 1% level = 2.638

Out of the 231 F_2_ plants, 47 highly tolerant and 47 most sensitive lines were selected, representing the two extreme tails of the phenotypic distribution, based on their responses to salt stress for the traits described below.

### Agronomic traits assessed under salt stress

#### Visual phenotypic score (SES score)

The two parents exhibited extreme responses to salt stress, with BRRI dhan29 (BR29) being highly sensitive (SES score = 9) and Capsule being tolerant (SES score = 4). Figure 1a shows the frequency distribution of SES scores among individuals of the mapping population and their parents. For the 47 selected tolerant F_2:3_ families, the final visual SES score ranged from 3 to 5, with an average of 4, while the final SES score for the 47 sensitive families ranged from 6.5 to 9 (average score = 7.18).

#### Survival

The mean value of survival for Capsule was 95% (range of 90–100%); while that of BR29 was 12% (range of 0–15%) (Fig. 1b). The frequency distribution of this trait seems to follow a bimodal distribution that is negatively skewed (skewness = − 0.65). About 17% of individuals in the population showed better survival (96–100%) than the donor parent, while only 2% were more sensitive than BR29.

#### Shoot length and shoot and root biomass

Mean shoot length for Capsule was 53.5 cm (range of 50–60 cm); while that of BR29 was 25.0 cm (range of 24–31 cm) (Fig. 1c). Variation in shoot length of their F_2:3_ progeny was also large (range of 18–67 cm). The frequency distribution also showed a bimodal distribution with a skewness value of − 0.35. Shoot biomass ranged from 19 to 2416 mg, with values of 1820 mg and 110 mg for Capsule and BR29, respectively (Fig. 1d). Out of the 94 F_2:3_ progenies, 53 individuals have shoot biomass in the range of 580–1820 mg and only 12 individuals had shoot biomass of about 120 mg or less, similar to that of BR29. This distribution is positively skewed (0.37). Transgressive segregation was observed in both directions (higher than the tolerant parent and lower than sensitive parent) for traits like survival and biomass under salt stress (Fig. 1b and d).

Root biomass was significantly reduced by salt stress, with the range of 31 to 450 mg (Fig. 1f). The root biomass production of the intermediate group (44 individuals) ranged from 172 to 266 mg. However, two extreme groups had low frequencies (10 plants with 31 to 78 mg; and 6 individuals with 407 to 450 mg). The distribution for root biomass was positively skewed (0.57).

### Physiological traits under salt stress

The lowest Na^+^ concentration (0.1 mmol g^− 1^dwt) was observed in 50 individuals and highest concentration (0.9 mmol g^− 1^ dwt) was in 7 individuals (Fig. 1f). The rest of the individuals had intermediate Na^+^ concentration. The parents Capsule (0.25 mmol g^− 1^ dwt) and BR29 (0.84 mmol g^− 1^ dwt) vary considerably in their Na^+^ concentration. The data is positively skewed (Skewness + 1.73), with a long right tail. Potassium concentration was also positively skewed (1.93). Around 49 individuals had low (0.01–0.10 mmol g^− 1^ dwt) K^+^ concentration, 40 plants had a range of 0.12–0.30 mmol g^− 1^ dwt. The parents Capsule (0.40 mmol g^− 1^ dwt) and BR29 (0.12 mmol g^− 1^ dwt) vary considerably in their K^+^ concentration. The Na^+^-K^+^ ratio of around 45 individuals of this population ranged from 1 to 3 and 40 progenies were intermediate with ratios of 6 to 14, with the rest having higher Na^+^-K^+^ ratios. The Na^+^-K^+^ ratio of Capsule and BR29 is approximately 2.3 and 12.0 respectively. The distribution is positive skewed with a skewness value of 1.5. SPAD readings were lower under salt stress and ranged from 0 to 38 (Fig. 1i). The SPAD reading of the intermediate group of 74 individuals ranged from 13 to 26. However, two extreme groups had low frequencies (6 plants had 0 to 9 SPAD reading; and 14 individuals with 31 to 38). Capsule (33) and BR29 (11) differ significantly in their SPAD readings. The data was positively skewed (0.57).

### Correlation analysis between traits in the mapping population

For complex traits like salinity, information on the relationship of the overall phenotypic performance and its contributing characters is a requisite to develop efficient selection strategies. Significant and positive correlations were observed among SES (Standard Evaluation System scores), Na^+^ concentration and Na^+^-K^+^ ratio (Table 1). Correlations between SES scores and survival, Na^+^ concentration and survival, Na^+^ concentration and shoot & root biomass, SES with shoot length, SPAD values and biomass were significant and negative. The positive correlations between SES and Na^+^ concentration indicates that visual stress scores were dependent on the levels of sodium accumulation in plant tissue. Additional parameters evaluated at the seedling stage included K^+^ concentration, biomass and SPAD readings. Na^+^ concentration and seedling growth and biomass seem to be the most important mechanisms associated with seedling survival under salt stress, as indicated by the high correlations between these parameters (Table 1).

### Path analysis of salt tolerance related agronomic and physiological traits

The different salt stress tolerance-related traits such as Na^+^ concentration and Na^+^-K ratio, had small direct effects on the overall tolerance (Table 2). The negative sign indicates the trait had a negative direct effect on the overall performance. Importantly, the lower value of SES score denotes higher tolerance and the lower value of Na^+^ concentration indicates lower SES score, which is in agreement with the correlation (0.69). This trait had an indirect effect on tolerance via survival. It is evident that the negative direct effect (− 0.31) of survival on SES is indicative of tolerance. The values of other traits like K^+^ concentration, Na^+^-K^+^ ratio, biomass, shoot length and SPAD readings are indirect path coefficients, and they reveal the degree to which these traits indirectly influenced SES scores via affecting other traits (Table 2). The summation of indirect path coefficients for each trait indicated the total value of indirect effects.

**Table 2:** Correlation and path coefficients for direct and indirect effects of Na^+^ concentration, survival, K^+^ concentration, Na^+^-K^+^ ratio, shoot and root biomass, shoot length and SPAD readings on SES scores in an F_2:3_ population of a cross between Capsule (salt tolerant) and BR29 (salt sensitive) grown under salt-stress at seedling stage

Predictor variables	Correlation	Direct effects (D)	Indirect effects (I)									Total effects (D + I)
			Na^+^ conc.	Survival	K^+^ conc.	Na^+^-K^+^ ratio	Biomass		Shoot length	SPAD	Total (I)	
							Shoot	Root				
Na^+^ concentration	0.692	0.147		0.220	−0.049	0.015	0.156	0.117	0.027	0.057	0.543	0.690
Survival	−0.795	−0.309	− 0.51		−0.056	− 0.010	0.171	0.138	−0.031	− 0.046	−0.485	− 0.794
K^+^ concentration	−0.184	− 0.104	− 0.121	0.061		− 0.11	0.04	0.048	0.003	0.005	−0.074	− 0.178
Na^+^-K^+^ ratio	0.440	0.024	0.035	0.093	0.035		0.110	0.091	0.017	0.034	0.415	0.439
Biomass (Shoot)	−0.815	−0.247	− 0.047	− 0.214	− 0.068	0.011		− 0.177	− 0.030	− 0.042	− 0.568	−0.815
Biomass (root)	−0.810	− 0.191	− 0.046	−0.223	− 0.061	− 0.012	0.230		−0.029	− 0.040	− 0.618	− 0.809
Shoot length	−0.739	− 0.040	−0.049	− 0.240	− 0.049	− 0.010	0.185	− 0.138		−0.047	− 0.699	−0.739
SPAD	−0.541	−0.092	− 0.046	−0.156	− 0.038	−0.010	0.114	−0.083	− 0.021		−0.449	− 0.541

Residual effect (R) = 0.43

The residual effect (R) determines how best the factors (independent variables) account for the variability of the dependent factor (salt tolerance), which is estimated as *R* = 0.43 (Table 2), so the variables Na^+^ concentration, K^+^ concentration, Na^+^-K^+^ ratio, survival, shoot length, biomass and SPAD value collectively explain 57% of the variability in salinity tolerance. Whereas the five traits, Na^+^ concentration, K^+^ concentration, survival, Na^+^-K^+^ ratio and shoot biomass control 50% of the variation (*R* = 0.50) in salt stress tolerance (Additional file 1: Table S1a). Na^+^ concentration, K^+^ concentration and survival accounted for approximately 40% (*R* = 0.61) of the total phenotypic variation (Additional file 1: Table S1b). The data suggest that the rest of the traits (Na^+^-K^+^ ratio, shoot biomass) are responsible for around 10% of the phenotypic variation for salinity tolerance.

### Marker polymorphism, segregation distortion and genetic linkage mapping

Four hundred and fifty microsatellite and InDel markers were used to screen the parents to identify polymorphic markers, and 105 markers (23.3%) were found to be polymorphic. The lower percentage of polymorphism is probably because both parents (Capsule and BR29) are of *indica* origin, and consequently, a relatively high degree of genetic similarity between them is expected. These polymorphic markers were then genotyped across the 94 F_2_ individuals. All the 105 markers exhibited clear banding patterns, with the amplified fragments ranged in size from 80 to 330 bp. The expected genotypic ratio in the F_2_ population would be 1:2:1 for homozygous BR29: heterozygous: homozygous Capsule and no segregation distortion occurred in 82 markers (Additional file 2: Table S2).

**Fig. 2:**
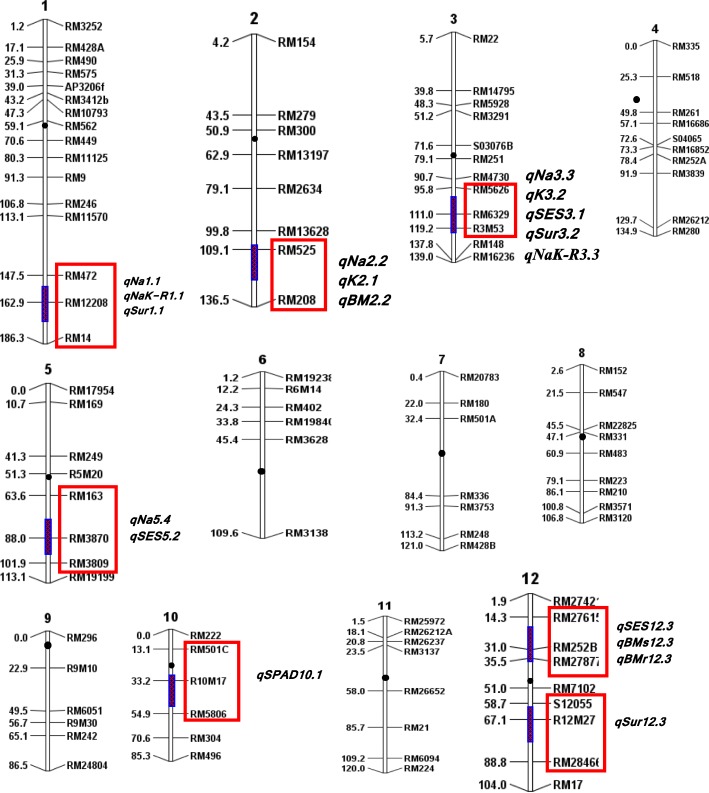
A genetic linkage map of the 12 chromosomes of rice constructed based on selected individuals of an F_2:3_ population of a cross between Capsule and BR29. The map was constructed using 105 SSR and InDel markers. The number at the top of each linkage group indicates the chromosome number. The names of the markers are listed at the right and the map distances between them (cM) are shown on the left of the chromosomes. Vertical bars on chromosomes 1, 2, 3, 5, 10 and 12 and the markers enclosed in red boxes indicate the approximate locations of the QTL detected for salinity tolerance

A molecular map was constructed using the Nipponbare/ Kasalath genetic map (Harushima et al. 1998) and the physical rice map (IRGSP (International Rice Genome Sequencing Project) and Matsumoto 2005) as references. The total size of the linkage map was 1442.2 cM (Kosambi mapping function: Kosambi, 1944) and the average interval size was 13.7 cM (Fig. 2). There were few gaps with large distances mainly located on chromosomes 2, 6, and 7. These gaps may indicate that the two parents are genetically more related at these regions, reducing the chances of finding polymorphic markers.

**Fig. 3:**
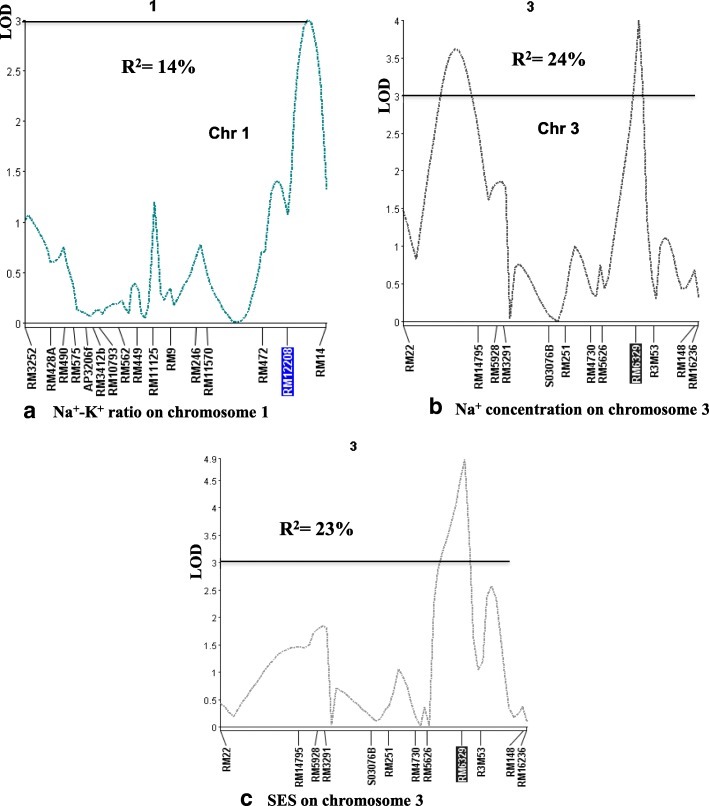
QTL likelihood curves of the LOD score of **a** Na^+^-K^+^ ratio on chromosome 1, **b** Na^+^ concentration and **c** overall phenotypic performance (SES) on chromosome 3, that were above the significance threshold of LOD = 3.0 and explained approximately 14%, 24% and 23% of the phenotypic variance for each trait

A total of 30 QTL were detected using single marker analysis and 20 of them were recognized by interval mapping and composite interval mapping at a LOD ≥ 3.0, while four other loci were just below the threshold between LOD = 2.8–2.9 (Table 3). QTL with large effect are illustrated on the molecular linkage map in Fig. 2. Representative QTL likelihood curves for newly identified loci for traits associated with seedling stage salt tolerance are shown in Fig. 3.

**Table 3:** QTL detected for salt tolerance-related traits viz. overall phenotypic performance (SES), survival, physiological and agronomic traits during seedling stage based on single-marker analysis (SMA), interval mapping (IM) and composite interval mapping (CIM) in the Capsule/BR29 F_2:3_ population

Traits	QTL	Chr.	Peak marker	Position (cM)	QTL bordering marker	Additive effect	^a^Direction of Phenotypic effect	SMA, IM or CIM	LOD	Threshold LOD values at α_0.05_ and α_0.01_ after 1000 iterations in permutation analysis	PVE (R^2^)
Agronomic traits											
SES	*qSES3.1*	3	RM6329	111.0	RM5626- R3M53	1.1	BR29	SMA	5.3		23.0
		3	RM6329	111.0	RM5626- R3M53	1.1	BR29	IM	5.3		23.0
		3	RM6329	111.0	RM5626- R3M53	1.1	BR29	CIM	4.8	4.0–5.07	21.0
	*qSES5.2*	5	RM3870	88.0	RM163-RM19199	1.2	BR29	SMA	3.7		17.0
		5	RM3870	88.0	RM163-RM19199	1.9	BR29	IM	3.7		17.0
		5	RM3870	88.0	RM163-RM19199	1.2	BR29	CIM	3.7		17.0
	*qSES12.3*	12	RM252B	31.0	RM27615-RM27877	1.0	BR29	SMA	2.9		17.0
Survival (%)	*qSur1.1*	1	RM12208	162.9	RM472-RM14	−10.6	Capsule	SMA	3.5		16.0
		1	RM12208	162.9	RM472-RM14	−10.6	Capsule	IM	3.9		18.0
		1	RM12208	162.9	RM472-RM14	−10.6	Capsule	CIM	4.0		17.0
	*qSur3.2*	3	RM6329	111.0	RM5626- R3M53	−20.1	Capsule	SMA	3.7		17.0
		3	RM6329	111.0	RM5626- R3M53	−20.1	Capsule	IM	3.7		18.0
		3	RM6329	111.0	RM5626- R3M53	−20.1	Capsule	CIM	3.7	3.7–4.9	18.0
	*qSur12.3*	12	R12M27	67.1	S12055-RM28466	−12.0	Capsule	SMA	2.8		14.0
		12	R12M27	67.1	S12055-RM28466	−12.5	Capsule	IM	3.1		16.0
Shoot length	*qSL1.1*	1	RM490	25.9	RM428A-AP3206f	−6.2	Capsule	SMA	3.4		16.0
	*qSL1.2*	1	RM12208	162.9	RM472-RM14	12.0	BR29	IM	3.2		15.0
		1	RM12208	162.9	RM472-RM14	10.0	BR29	CIM	3.0		14.2
	*qSL5.3*	5	RM3870	88.0	RM163-RM19199	−3.69	Capsule	SMA	3.9		18.0
		5	RM3870	88.0	RM163-RM19199	−3.69	Capsule	IM	4.5		20.0
		5	RM3870	88.0	RM163-RM19199	−4.50	Capsule	CIM	4.4	3.81–5.2	20.0
Biomass-shoot	*qBMs3.1*	3	RM6329	111.0	RM5626- R3M53	− 261.0	Capsule	SMA	3.0		13.0
		3	RM6329	111.0	RM5626- R3M53	−261.0	Capsule	IM	3.0		13.0
		3	RM6329	111.0	RM5626- R3M53	−261.0	Capsule	CIM	3.0		13.0
	*qBMs5.2*	5	RM3870	88.0	RM163-RM19199	−32.1	Capsule	SMA	4.1		18.0
		5	RM3870	88.0	RM163-RM19199	520.0	BR29	IM	4.2		19.0
		5	RM3870	88.0	RM163-RM19199	500.0	BR29	CIM	3.8		17.0
	*qBMs12.3*	12	RM252B	31.0	RM27615-RM27877	410.0	BR29	SMA	3.8		16.0
		12	RM252B	31.0	RM27615-RM27877	410.0	BR29	IM	3.4		16.0
Biomass-root	*qBMr1.1*	1	RM12208	162.9	RM472-RM14	48.0	BR29	SMA	3.5		17.0
		1	RM12208	162.9	RM472-RM14	42.0	BR29	IM	4.4		20.0
		1	RM12208	162.9	RM472-RM14	70.0	BR29	CIM	4.4		16.0
	*qBMr3.2*	3	RM6329	111.0	RM5626- R3M53	−31.0	Capsule	SMA	3.6		17.0
		3	RM6329	111.0	RM5626- R3M53	−42.0	Capsule	IM	3.5		16.0
		3	RM6329	111.0	RM5626- R3M53	−60.0	Capsule	CIM	3.5		15.0
	*qBMr12.3*	12	RM252B	31.0	RM27615-RM27877	58.0	BR29	SMA	3.5		15.0
		12	RM252B	31.0	RM27615-RM27877	90.0	BR29	IM	3.3		15.0
Physiological traits											
Na^+^ concentration	*qNa1.1*	1	RM12208	162.9	RM472-RM14	−0.30	Capsule	IM	3.0		12.0
	*qNa2.2*	2	RM525	109.0	RM525-RM208	−0.20	Capsule	SMA	3.2		15.0
		2	RM525	109.0	RM525-RM208	−0.20	Capsule	IM	3.2		14.0
	*qNa3.3*	3	RM6329	111.0	RM5626-R3M53	0.12	BR29	SMA	4.7		21.0
		3	RM6329	111.0	RM5626-R3M53	0.10	BR29	IM	4.4		20.0
		3	RM6329	111.0	RM5626-R3M53	0.10	BR29	CIM	4.3	4.2–5.5	24.0
	*qNa5.4*	5	RM3870	88.0	RM163-RM3809	0.25	BR29	IM	2.8		13.0
K^+^ concentration	*qK1.1*	1	RM12208	162.9	RM472-RM14	−0.10	Capsule	SMA	4.6		30.0
		1	RM12208	162.9	RM472-RM14	− 0.10	Capsule	IM	4.5		26.0
		1	RM12208	162.9	RM472-RM14	−0.10	Capsule	CIM	4.5	4.1–5.7	25.0
	*qK3.2*	3	RM6329	111.0	RM5626-R3M53	−0.10	Capsule	SMA	4.5		20.0
		3	RM6329	111.0	RM5626-R3M53	−0.10	Capsule	IM	4.5		20.0
		3	RM6329	111.0	RM5626-R3M53	−0.10	Capsule	CIM	4.5		16.0
	*qK12.3*	12	RM252B	31.0	RM27615-RM27877	−0.10	Capsule	SMA	3.5		15.0
		12	RM252B	31.0	RM27615-RM27877	−0.10	Capsule	IM	3.1		17.0
		12	RM252B	31.0	RM27615-RM27877	−0.10	Capsule	CIM	5.9		15.0
Na-K ratio	*qNaK-R1.1*	1	RM12208	162.9	RM472-RM14	1.5	BR29	IM	3.1		15.0
		1	RM12208	162.9	RM472-RM14	2.5	BR29	CIM	3.0		14.0
	*qNaK-R2.2*	2	RM525	109.1	RM3628-RM208	−2.8	Capsule	SMA	3.5		17.0
		2	RM525	109.1	RM3628-RM208	−2.5	Capsule	IM	4.5		16.0
		2	RM525	109.1	RM3628-RM208	−2.5	Capsule	CIM	3.6		17.0
	*qNaK-R3.3*	3	RM6329	111.0	RM5626- R3M53	−1.8	Capsule	SMA	4.4		22.0
		3	RM6329	111.0	RM5626- R3M53	−2.3	Capsule	IM	3.7		17.0
		3	RM6329	111.0	RM5626- R3M53	−2.5	Capsule	CIM	3.7		17.0
	*qNaK-R5.4*	5	RM3870	88.0	RM163-RM19199	1.4	BR29	IM	3.1		15.0
		5	RM3870	88.0	RM163-RM19199	1.3	BR29	CIM	3.2		15.0
SPAD readings	*qSPAD10.1*	10	R10M17	33.2	RM501C-RM5806	−5.3	Capsule	IM	3.2		15.0
		10	R10M17	33.2	RM501C-RM5806	−5.3	Capsule	CIM	3.2		15.0

^a^QGene calculates both LOD and additive effect and displays the LOD. If we select a signed statistic such as **Add effect** (additive effect), a second plot is drawn below the main chromosome-map plot (LOD curve). A positive-signed effect represents an increasing allele from parent1, BR29; a negative-signed effect, an increasing allele from parent 2, Capsule (i.e. the favorable allele comes from Capsule)

### QTL for agronomic traits associated with salinity tolerance

Three QTL, *qSES3.1, qSES5.2* and *qSES12.3,* were identified for visual symptoms using SES scores that explains 23.0, 17.0 and 17.0% of the phenotypic variance, respectively. The Capsule allele increased the overall phenotypic performance and reduced SES visual scores at all three loci. Three significant QTL were identified for survival on chromosomes 1, 3 and 12. For *qSur1.1*, the Capsule alleles had a positive effect on survival rate and explained 18.0% of the phenotypic variation. This QTL co-localizes with two other large effect QTL (*qNa1.1* and *qNaK-R1.1*) on the long arm of chromosome 1. Two QTL *qSur3.2* and *qSur12.3* were located between RM5626-RM148 and S12055-RM28466 with R^2^ values of 18.0% and 16.0%, respectively, with positive effects of the Capsule alleles for both QTL.

Three QTL were detected on chromosomes 1 and 5 that are significantly associated with shoot length. Capsule alleles contributed to taller shoots at all three loci. Two of these QTL, *qSL1.1* and *qSL1.2* were located between RM428A-AP3206f on the short arm of chromosome 1 and RM472-RM14 on the long arm, with R^2^ values of 16.0% and 15.0%, respectively. The third QTL on chromosome 5 was located between markers RM163-RM19199. Five regions were identified on chromosomes 1, 2, 3, 5 and 12, with significant LOD scores for shoot biomass and with positive effects of the alleles contributed by BR29 for this trait. These QTL also co-localize with other QTL in the same regions. Both interval mapping and composite interval mapping detected genomic regions associated with root biomass, with four significant QTL on chromosomes 1, 2, 3 and 12 near RM12208, RM525, R3M53 and RM252B, which, respectively, explain about 20.0%, 16.0%, 17.0% and 15.0% of the phenotypic variation; BR29 alleles had positive effect on root biomass. The minimum to largest threshold LOD (at α_0.05_ and α_0.01_) values ranged from 3.7–5.7 after 1000 iterations in permutation tests with their corresponding agronomic (SES-LOD value 4.8, survival- LOD: 3.7 and shoot length- LOD:4.4) and physiological (Na^+^ concentration- LOD: 4.3, K^+^ concentration- LOD:4.5) traits (Table 3).

### QTL for physiological traits

Interval mapping and composite interval mapping identified four QTL (*qNa1.1*, *qNa2.2*, *qNa3.3* and *qNa5.4*) on the long arms of chromosomes 1, 2, 3 and 5 that are significantly associated with Na^+^ concentration in plant tissue. Two QTL (*qNa1.1*, *qNa3.3*), respectively, accounted for about 12% and 24% of the total variation in Na^+^ concentration. Moreover, two additional QTL were detected through interval mapping, one each on chromosomes 2 and 5, and they explained 15% and 13% of phenotypic variation. Three significant QTL were detected through single marker analysis, interval mapping and composite interval mapping for K^+^ concentration. These QTL are on the long arm of chromosomes 2 and short arms of 3 and 12, between RM525-RM208, RM5626-R3M53 and RM27615-RM27877, accounting for about 30%, 20% and 15% of the total variation in K^+^ concentration, respectively. Capsule contributed the positive alleles for these QTL.

Both interval mapping and composite interval mapping detected one significant QTL for Na^+^-K^+^ ratio on chromosome 1 near RM12208, controlling 15% of the phenotypic variation; and BR29 allele had positive effect in increasing Na^+^-K^+^ ratio. Three QTL with significant LODs were also identified on chromosomes 2, 3 and 5 for this trait, with R^2^ values of 17%, 22% and 15%, respectively. Based on SPAD readings, one QTL was identified that was associated with chlorophyll content, with a significant LOD score, and with Capsule as the source of the positive allele. The QTL was located on chromosome 10, and explained 15% of the phenotypic variation for this trait.

Multilocus analysis indicated that the QTL identified on different chromosomes were, for most part, independent of each other, and that their effects were generally additive. For example, the major QTL for Na^+^ concentration on chromosomes 1 (interval RM472-RM14, with PVE of 12%), 3 (RM5626-R3M53, PVE 20%), and 5 (RM163-RM3809, PVE 13%) could together account for 45% of the total phenotypic variation. Likewise, the combined effect of the QTL *qSES3.1* (*R*^*2*^ = 23%), *qSES5*.*2* (*R*^*2*^ = 17%), and *qSES12.3* (*R*^*2*^ = 17%) was responsible for 57% of the total phenotypic variation (Table 3).

### Distributional-extreme analysis

The results of distributional-extreme analysis were generally consistent with the results of single-marker analysis, interval mapping and composite interval mapping. Monogenic segregation of SSR and InDel markers, which were identified to be QTL-linked by single-marker analysis, interval mapping and/or composite interval mapping are shown in Table 4 for the selected salt-tolerant and salt-sensitive classes. The results indicated presence of large allele frequency differences between the extreme classes for these markers, suggesting their association with the QTL affecting salt tolerance during seedling stage in rice. However, in most cases the marker-allele frequency differences were only close to the significant level, but were significant (*P <* 0.05) for markers on chromosomes 1, 2, 3, 5, 10 and 12, where major QTL have been identified (Table 4).

**Table 4:** Segregation of SSR and InDel markers that exhibited association with QTL for salinity tolerance in selected salt-tolerant and salt-sensitive classes in an F_2_ population of a cross between Capsule (tolerant) and BR29 (sensitive) genotypes. A negative sign of the allele frequency difference between the tolerant and sensitive classes (*Pr-Ps*) indicates allele-frequency changes in the opposite direction to the parental phenotype

Genetic Marker	Tolerant class				Sensitive class				Difference		Confidence interval > 95% i.e. *(Pr-Ps) ≥ 2*σ_*p*_	*R*^*2*^_p_ (%)
	E/E^a^	E/Cap	Cap	*Pr* ^*b*^	E/E	E/Cap	Cap	*Ps* ^*b*^	*Pr-Ps*	Standard error (σ_p_)		
Chromosome 1												
RM472	14	21	12	0.223	10	23	14	0.245	−0.021	0.0617	0.123	–
RM12208	10	33	4	0.351	13	18	16	0.191	0.160^*^	0.0638	0.128	12.66
RM14	3	33	11	0.351	15	17	15	0.181	0.170^*^	0.0632	0.126	15.05
Chromosome 2												
RM525	17	21	9	0.223	12	26	9	0.277	−0.053	0.0630	0.126	–
RM208	17	20	10	0.213	9	21	17	0.223	−0.011	0.0602	0.120	–
Chromosome 3												
RM4730		25		0.266		38		0.404	−0.138^*^	0.0681	0.136	6.11
RM5626	7	25	15	0.266	15	24	8	0.255	0.011	0.0640	0.128	–
RM6329	5	32	10	0.340	20	19	8	0.202	0.138^*^	0.0641	0.128	9.13
R3M53	15	21	11	0.223	10	33	4	0.351	−0.128^*^	0.0653	0.131	5.50
Chromosome 5												
RM3870	10	19	18	0.202	11	33	3	0.351	−0.149^*^	0.0643	0.129	7.49
RM3809	13	23	11	0.245	10	26	11	0.277	−0.032	0.0640	0.128	–
RM19199	19	17	11	0.181	14	20	13	0.213	−0.032	0.0579	0.116	–
Chromosome 10												
RM501C	10	26	11	0.277	16	23	8	0.245	0.032	0.0640	0.128	–
R10M17	12	22	13	0.234	16	21	10	0.223	0.011	0.0613	0.123	–
RM5806	11	26	10	0.277	12	28	7	0.298	−0.021	0.0660	0.132	–
Chromosome 12												
RM27615	20	22	5	0.234	12	27	8	0.287	−0.053	0.0639	0.128	–
RM252B	20	23	4	0.245	2	36	9	0.383	−0.138^*^	0.0669	0.134	6.23
RM27877	6	32	9	0.340	15	28	4	0.298	0.043	0.0679	0.136	–
S12055	6	31	10	0.330	15	29	3	0.309	0.021	0.0680	0.136	–
R12M27	10	33	4	0.351	5	27	15	0.287	0.064	0.0678	0.136	–
RM28466	11	22	14	0.234	14	21	8	0.223	0.011	0.0613	0.123	–

^*^Significant at *P <* 0.05

^a^E/E = homozygous for BR29 alleles; E/Cap = heterozygous; ^b^Capsule allele frequency among the tolerant *(Pr)* and sensitive progeny *(Ps); R*^*2*^_p_ = phenotypic variation explained

The phenotypic variation (*R*^*2*^_p_) explained by these QTL was relatively consistent (Table 4) with the PVE estimated using single-marker analysis, interval mapping and composite interval mapping (Table 3). The observation that no other marker locus (Additional file 3: Table S3) in this population exhibited a marker-allele frequency difference as large as that observed for any of the markers in Table 4, indicates that significant markers were most likely linked to QTL affecting salt tolerance.

### Epistatic interaction

A two-way test to detect epistatic interactions (*P* ≤ 1.0 × 10^− 5^) between marker loci was performed using the Map Manager QTXb20 software. The analysis identified six interactions consisting of 4 markers on chromosome 3 (Additional file 4: Table S4). Out of these, one marker (RM6329) contributed the main effect QTL and also had a significant effect on the final phenotype (SES and survival) combined with other makers, indicating strong interactions between these QTL and background loci of chromosome 3. Two QTL (*qSur3.2* and *qBMs 3.3*) detected on chromosome 3 were located in the same region at 119.2 cM between RM5626 and RM148 that was associated with salt tolerance, which originated from BR29.

### Relationships between phenotypes and QTL genotypes

The correspondence between the phenotypes and QTL genotypes of the F_2:3_ families were determined by graphical genotyping (Young and Tanksley 1989). Graphical genotyping of extreme F_2_ plants indicated that they contained all or most of the identified QTL for salinity tolerance. This is shown for the five most tolerant and five most sensitive F_2_ plants for chromosomes 1 and 3 (Additional file 5: Figure S1). It is noteworthy that all five tolerant individuals had the favorable QTL alleles originating from the salt tolerant parent Capsule, in either the homozygous or the heterozygous form at the QTL location in both chromosomes (marker RM12208 for chromosome 1). The five sensitive individuals had the negative alleles in homozygous form for the sensitive parent BR29 at this location in both chromosomes (marker RM6329, on chromosome 3).

## Discussion

### Responses of the selected F_2:3_ progeny and their parental lines to salt stress and path coefficient analysis

The major traits- SES scores, survival, shoot biomass, Na^+^ concentration, K^+^ concentration and Na^+^-K^+^ ratio seem to play crucial roles in salt tolerance. SES scores of the tolerant set ranged from 3 to 5, with an average of 4; while that of the sensitive set ranged from 6.5 to 9. The tolerant individuals had the lowest Na^+^ concentration (0.1 to 0.3 mmol g^− 1^ dwt); while the sensitive individuals had the highest (0.9 mmol g^− 1^ dwt) and consequently, higher Na^+^-K^+^ ratios. Accumulation of toxic elements like sodium in plant tissue is known to affect plant growth through disrupting protein synthesis and interfering with enzyme activity, causing premature senescence of older leaves, and ultimately hindering overall growth and reducing grain yield (Bhandal et al. 1988; Blaha et al. 2000; Munns 2002; Tester and Davenport 2003; Munns et al. 2006; Munns and Tester 2008). Rapid biomass accumulation through faster growth at early seedling stage could dilute Na^+^ concentration in plant tissues.

The negative association between SES scores with survival, Na^+^ concentration and survival, Na^+^ concentration and shoot & root biomass, SES with shoot length, SPAD values, and biomass clearly demonstrated the detrimental effects of high Na^+^ accumulation in plant tissue under saline conditions. Damaging effects of salinity on growth and yield of rice were reported before (Flowers and Yeo 1981, 1995; Yeo and Flowers 1982; Gregorio and Senadhira 1993; Ashraf et al. 1999; Moradi 2002; Flores 2004; Thapa 2004; Ismail et al. 2007; Munns and Tester 2008; Ding et al. 2010; Ismail and Horie 2017). The information on associations among variables showed the nature and extent of the relationship, as well as the interdependencies of these traits. This information will help in simultaneous selection of different traits that contribute to important complex characters such as salt tolerance and grain yield, in a breeding program. The significant and positive correlation between SES and Na^+^ concentration also suggested that the overall phenotypic response was substantially influenced by Na^+^ concentration as reported before (Moradi et al. 2003; Moradi and Ismail 2007; Ismail et al. 2007; Collins et al. 2008; Munns and Tester 2008; Platten et al. 2013).

However, associations detected from correlation coefficients may not necessarily be attributed to a single variable but rather to a number of interdependent variables. Therefore, decisions based solely on correlation coefficients may not necessarily be reliable, as only limited information is revealed about what may essentially be a complex series of interrelationships between variables (Kang 1994). We therefore used path analysis to dissect the correlation coefficients into direct and indirect effects to quantify the relative contribution of each component trait to the overall correlation (Yao et al. 2002; Rebetske et al. 2002; Condon et al. 2004).

Based on this analysis, the different salt tolerance traits (causal factors) such as Na^+^ concentration, K^+^ concentration, Na^+^-K^+^ ratio, survival, shoot length, biomass and SPAD value contributed to the overall salt tolerance (effect). Therefore, this overall salt tolerance is the result of these seven traits and some other un-investigated traits (unidentified factors or residual effect: R), which agrees with previous studies (Sidewell et al. 1976; Gravois and Helms 1992; McGiffen et al. 1994; Sokal and Rohlf 1995; Board et al. 1997).

A path coefficient is a measure without dimension and can eliminate the contributions of different variances to a physiological trait; the implementation of path analysis can objectively evaluate the relative importance of every trait to salt tolerance (He et al. 2003; Munns 2005), represented by SES in this study. In this investigation, path analysis showed that Na^+^ concentration, K^+^ concentration, Na^+^-K^+^ ratio and survival are the most important traits that determine the extent of salinity tolerance because these traits collectively explain 46% of the phenotypic variation. Several researchers also made similar observations (Yeo and Flowers 1986; Yeo et al. 1990; Peng and Ismail 2004; Ismail et al. 2007; Collins et al. 2008; Rahman et al. 2016; Ismail and Horie 2017). Thus selection based on these characters should favor the development of highly tolerant varieties combining the most important characters. As an analytical tool, path analysis has potential for application in breeding for traits that are genetically complex, like salt tolerance, as it allows separating and quantifying closely associated variables. Hence, correlation coefficients and their partitioning into direct and indirect effects would provide useful information for detection of mechanisms that are relatively more crucial for seedling-stage salinity tolerance, and hence, planning a successful breeding program based on those traits.

### Marker segregation and genomic regions important for salinity tolerance

The present result was supported by the typical Mendelian segregation for F_2_ population in 82 SSRs and InDel markers at a probability of *P >* 0.05 to fit the null hypothesis of no difference between the expected and observed results. Furthermore, relatively less segregation distortion was observed in the remaining 23 markers at a significance level of *P* < 0.05 and *P* < 0.01, as the deviation expected from Mendelian segregation ratios.

A total of 27 significant QTL regions were identified in the present study, and are associated with different growth and physiological traits affecting salinity tolerance. Four QTL (*qNa1.1, qNaK-R1.1, qK2.1 and qSES3.1*) are noteworthy, since they detect overlapping agronomic and physiological traits associated with salinity tolerance. Four QTL, *qNa2.2* and *qNaK-R2.2* on chromosome 2 and *qSES12.3* and *qSur12.3* on chromosome 12 explained over 32% and 25% of the pooled phenotypic variations, respectively. These QTL are now the focus for more detailed analysis to get better insights into their potential roles in physiological processes affecting salt tolerance in rice, and also for use in breeding. This functional understanding may also be important to identify and validate candidate genes underlying these QTL after their fine mapping.

It was observed in multi-locus analysis that, if the combined effects of some major QTL (*qNa1.1, qNa3.3*, and *qNa5.4*) for Na^+^-concentration on different chromosomes such as 1, 3, 5 (pooled PVE of three Na^+^-concentration QTL is 45.0%; with R^2^ = 12%; 20% and 13%, respectively, and similarly SES scores (pooled PVE of three SES QTL is 57% with R^2^ values of 23% for *qSES3.1*, and 17% for each of *qSES5*.*2* and *qSES12.3*) when major QTL of both traits were considered simultaneously, their pooled effects were less than the overall summation of their individual effects. This is probably because of i) co-localization of the QTL, ii) less-than-additive epistatic interactions among QTL (Eshed and Zamir 1996), or iii) some of the QTL affect this trait through similar pathways.

### Identification of marker-linked QTL through analysis of distributional extremes and pleiotropy

QTL were detected by applying bidirectional trait-based selective genotyping, which is more robust and reliable (Zhang et al. 2003; Navabi et al. 2009) than unidirectional selective genotyping where the allele frequencies from sensitive class are not considered (Stuber et al. 1980; Falconer 1989; Lander and Botstein 1989; Foolad and Jones 1993; Foolad et al. 2001). Bidirectional trait-based marker analysis is used to detect the significant difference between the frequency of favorable alleles in the selected high class (e.g., salt tolerance) and the frequency of alleles of the low class (e.g., salt sensitivity) comparing confidence intervals for declaring QTL of interest. Here, selection for salinity tolerance/sensitivity resulted in significant allele frequency differences between the two selected classes at several marker loci on chromosomes 1, 3, 5, and 12, suggesting the presence of QTL for salinity tolerance on these chromosomes. At each marker locus, a significant allele frequency difference between the salt tolerant and salt sensitive classes was inferred as an association of the marker locus with a putative QTL affecting salt tolerance (Stuber et al. 1980; Lebowitz et al. 1987; Lander and Botstein 1989; Foolad et al. 1997).

For quantitative traits like salinity tolerance, however, if the marker loci are associated with the QTL either directly due to pleiotropic effects or more likely, due to physical linkage, the marker allele frequencies will also change in response to selection. In this study, the QTL *qNa3.3, qK3.2, qSES3.1, qNaK-R3.3* and *qSur3.2* corresponding to genetic marker RM6329 with *R*^*2*^p of 9.13% were co-localized on chromosome 3, and *qNa1.1, qSur1.1, qNaK-R1.1* corresponding to marker RM12208 with *R*^*2*^p of 12.66% also shared a common genomic region on the long arm of chromosome 1 (Table 4) are hotspots for salinity tolerance. The other significant markers like RM14 on chromosome 1, RM4730, RM5626 and R3M53 on 3; RM3870 on 5 and RM252B on chromosome 12 are linked with main effect QTL. These QTL clusters might have pleiotropic effects or are physically linked. Thus, any significant change in marker allele frequencies in response to selection can be attributed to the association of marker loci with QTL affecting the trait under selection (Stuber et al. 1980; Lebowitz et al. 1987; Lander and Botstein 1989; Darvasi and Soller 1992; Foolad and Jones 1993). Several chromosomal regions were associated with more than one trait, indicating linkage or pleiotropic effects. For instance, the QTL *qNa1.1, qNaK-R1.1*, *qK1.1, qSur1.1* and *qBMr1.1* linked with RM12208, are, respectively, responsible for low Na^+^ concentration, low Na^+^-K^+^ ratio, high K^+^ concentration, and high % survival and root biomass, were all located in the same region on the long arm of chromosome 1, conferring salt tolerance. It is important to point out that the same QTL might contribute to several traits associated with a specific phenotype. Hence, epistatic effects and pleiotropy can play a notable role in the interaction and function of a QTL. A QTL with small effect might have a large effect in a regulatory pathway (Koyama et al. 2001). In this study, many main-effect QTL other than epistasis for different traits were detected in the same or near regions, suggesting there are possible pleiotropic effects on different related traits in the said regions.

Potential epistatic interactions between marker loci identified 4 markers resulting in 6 two-way interactions using MapManager QTX software (Additional file 4: Table S4). This suggests that these epistatic interactions are important components of the genetic basis of salt stress tolerance. Individually, all these markers had no effect on the trait except for marker RM6329, but resulted in an enhanced effect when combined with another marker. Assessing the importance of epistatic interactions is crucial because epistasis complicates the genotype-phenotype relationships, and competing models of evolution differ largely in determining the importance of epistasis (Malmberg et al. 2005).

Transgressive segregation was observed in several traits in this population; and could be attributed to several factors; i) epistasis (Additional file 4: Table S4), and/or ii) the action of complementary genes. Quantitative genetics studies of breeding populations consistently point to the action of complementary genes as the primary cause and more accepted explanation for transgressive segregation, although epistasis and overdominance may also contribute to it (Grant 1975; Vega and Frey 1980; Rieseberg et al. 1999).

These results indicate that the QTL identified in this study are genuine and thus could be pyramided to improve salt tolerance during seedling stage in rice, especially since some of these QTL are novel. The tolerant individuals exhibited tolerance even when the QTL regions are in the heterozygous condition (Additional file 5: Figure S1) suggesting that these QTL may have dominant effects. Furthermore, the results of the graphical genotyping reveal the power of marker analysis for precise identification of genomic locations with significant effects on quantitative traits and thus, the predictive value of QTL genotyping (Foolad et al. 1998).

### Comparison of the new QTL with previously mapped QTL

The QTL identified in this study were compared with those identified in earlier studies as being associated with salinity tolerance at various growth stages (Additional file 6: Table S5; Additional file 7: Figure S2). The major QTL, *Saltol,* derived from the salt tolerant landrace Pokkali, has been mapped on chromosome 1, conferring salt tolerance at vegetative stage, with strong phenotypic effect (R^2^ of 39% to 44%) (Bonilla et al. 2002; Kim et al. 2009; Thomson et al. 2010). Another gene, *SKC1*, was also identified at this locus for salt tolerance during vegetative stage in the cultivar Nona Bokra (Ren et al. 2005). Both *Saltol* (10.6–11.5 Mb) and *SKC1* (11.46 Mb) were absent in Capsule. Three new co-located QTL (*qNa1.1, qNaK-R1.1, qSur1.2*) on chromosome 1 and four QTL (*qNa3.3, qNaK-R3.1, qSur3.2* and *qSES3.1*) on 3 were identified in 162.9 cM and 111 cM positions, respectively. Stacking these new QTL with *Saltol* or *SKC1* might result in much higher tolerance.

Several studies have reported QTL for traits related to salinity tolerance on chromosomes 1, and 7 (Zhang et al. 1995; Gong et al. 1999) and for seedling survival under saline conditions on chromosome 5 (Lin et al. 2004). The three QTL (*qSur1.1, qSur3.2* and *qSur12.3*) identified for survival on chromosomes 1, 3 and 12 in this study were at different genomic locations. We summarized the QTL positions identified in this study as compared with those identified in previous studies (Flowers et al. 2000; Prasad et al. 2000; Koyama et al. 2001; Bonilla et al. 2002; Lin et al. 2004; Ren et al. 2005; Lee et al. 2006; Ghomi et al. 2013; Bimpong et al. 2014; Bizimana et al. 2017; De Leon et al. 2017) in Additional file 6: Table S5. None of those QTL have the same map locations with the QTL identified here except for *qSL1.1* (De Leon et al. 2017) and *qSL1.2* (Bizimana et al. 2017; Additional file 6: Table S5), suggesting that most of these QTL are novel and candidates for use in breeding.

Several QTL with large effects were reported on chromosomes 1, 3 and 5 (Fig. 3). One of these QTL for Na^+^ concentration had an LOD of 3.2 and R^2^ of 15% in interval mapping. Another three QTL were identified on chromosome 1 for Na^+^-K^+^ ratio and K^+^ concentration. These QTL co-localize at the same position (RM12208 marker), suggesting functional relatedness. This major QTL might also have pleiotropic effects on other traits. The cluster of QTL on chromosome 1 for different traits (Fig. 2) was also supported by the strong correlations observed among these traits (Table 1), and could be attributed to linkages and/or pleiotropic effects. High-resolution mapping is required to determine whether pleiotropic effects are present. QTL analysis with selective genotyping is limited to one trait (Bernardo 2002). In this study, low-cost trait-based selective genotyping has been applied on lines selected for more than one trait, suggesting the detected QTL seem to influence multiple traits with adaptive values for salinity tolerance.

A summary of major QTL identified before and also in this study is shown in Additional file 7: Figure S2. Three major loci for Na^+^ concentration (*qNa1.1*), Na^+^-K^+^ ratio (*qNaK-R1.1*) and survival (*qSur1.1*) (Additional file 7: Figure S2D) and a cluster of large effect QTL identified on chromosome 3 for SES (*qSES3.1*), Na^+^ concentration (*qNa3.3*) and survival (*qSur3.2*), which is also considered novel (Additional file 7: Figure S2E) should be targeted for pyramiding through marker assisted breeding. Since several QTL were identified for each trait in this study, and some of the identified QTL explained a high phenotypic variance, these loci could subsequently be used for QTL pyramiding. The use of three approaches for QTL detection and then comparing the results with the bidirectional distributional extreme analysis is statistically more powerful and reproducible (Navabi et al. 2009).

## Conclusions

The overall phenotypic performance under salt stress reflected by visual SES scores is determined by several key traits, including survival, sodium and potassium concentration and rate of growth. Here we used path analysis to assess the genetic contribution of different mechanisms of salinity tolerance during early vegetative stage in the rice landrace Capsule, to identify key factors associated with salt tolerance. The results suggest that selection should be made based on Na^+^ and K^+^ concentrations and ratios in plant tissue, and seedling survival to fast track the development of improved salt tolerant varieties. Most of the QTL identified here through single marker analysis were also detected using interval mapping and composite interval mapping, and were further confirmed through graphical genotyping. Several QTL were identified on chromosomes 1 (*qNa1.1*, *qK1.1*, *qNaK-R1.1*, *qSur1.1*), 2 (*qNa2.2*), 3 (*qSES3.1, qNa3.3, qK3.2, qNaK-R3.3, qSur3.2*), 5 (*qSES5.2, qNa5.4, qNaK-R5.4*) and 12 (*qSES12.3, qK12.3, qSur12.3*), that are associated with tolerance at seedling stage, and the newly mapped loci on chromosomes 1 and 3 are novel. These QTL are good targets for subsequent fine mapping and cloning to develop gene-based SNP markers. Pyramiding these QTL with previously identified loci will help develop highly tolerant varieties for salt affected areas, especially coastal areas where salt stress is a major impediment for rice production during both dry and wet seasons; an effect further worsening with climate change.

## Additional files

Additional file 1: **Table S1a.** Correlation and path coefficients for direct and indirect effects of Na^+^ uptake, K^+^ uptake, survival, Na^+^-K^+^ ratio, and shoot biomass on SES scores in an F_2:3_ population of a cross between Capsule (salt tolerant) and BR29 (salt sensitive) grown under salt-stress at seedling stage. **Table S1b.** Correlation and path coefficients for direct and indirect effects of Na^+^ uptake, survival and K^+^ uptake on SES scores in an F_2:3_ population of a cross between Capsule (salt tolerant) and BR29 (salt sensitive) grown under salt-stress at seedling stage. (PDF 843 kb)

Additional file 2: **Table S2.** χ2-test statistics testing of markers showing Mendelian inheritance for goodness of fit. (PDF 941 kb)

Additional file 3: **Table S3.** Segregation of SSR and InDel markers that were not associated with QTL for salinity tolerance in selected salt-tolerant and salt-sensitive classes in an F_2_ population of a cross between Capsule (salt tolerant) and BR29 (salt sensitive). (PDF 848 kb)

Additional file 4: **Table S4.** Significant digenic/ epistatic interactions (*P* ≤ 1.0 × 10^− 5^) and LOD values for agronomic and physiological traits in an F_2:3_ population from a cross between Capsule/BR29. (PDF 843 kb)

Additional file 5: **Figure S1.** Chromosomal constitution of five salt-tolerant and five salt-sensitive F_2_ individuals for chromosome 1 and 3. The sky blue area indicates heterozygous genotypes at the marker loci, the green area indicates homozygous donor parent genotypes and the red color area indicate sensitive parent BR29 alleles. The names of markers are listed at the top of the figure. It is apparent that the salt tolerant individuals had the favorable QTL alleles (homozygous or heterozygote alleles from Capsule) and the salt sensitive individuals had opposite QTL alleles from BR29 at the QTL loci (RM12208 and RM6329). (PDF 843 kb)

Additional file 6: **Table S5.** Comparisons of the QTL regions identified in this study for salinity tolerance at seedling stage, with previously mapped QTL from different populations and for different growth stages. (PDF 845 kb)

Additional file 7: **Figure S2.** Summary of three major loci identified in this study. A, B, and C refer to the *Saltol* region on the short arm of chromosome 1, which was also identified before (Bonilla et al. 2002; Lin et al. 2004; Ren et al. 2005), whereas D and E represent two novel genomic regions identified in this study on the long arms of chromosome 1 and 3, respectively. (PDF 843 kb)

## Data Availability

The datasets supporting the conclusions of this article are included within the article and its additional files.

## Abbreviations

CIM: Composite interval mapping
GGT: Graphical genotyping
IM: Interval mapping
LOD: Logarithms of odds
PVE: Phenotypic variation explained
SD: Segregation distortion
SES: Standard evaluation system
SMA: Single-marker analysis
TBA: Trait-based marker analysis
