# Supplementary material for: Assessing trait contribution and mapping novel QTL for salinity tolerance using the Bangladeshi rice landrace Capsule
Source: Rice (N Y). 2019 Aug 13;12:63. doi: 10.1186/s12284-019-0319-5 (PMC6692794; doi:10.1186/s12284-019-0319-5)
Supplement: Supplementary file 1 — Table S1a. Correlation and path coefficients for direct and indirect effects of Na+ uptake, K+ uptake, survival, Na+-K+ ratio, and shoot biomass on SES scores in an F2:3 population of a cross between Capsule (salt tolerant) and BR29 (salt sensitive) grown under salt-stress at seedling stage. Table S1b. Correlation and path coefficients for direct and indirect effects of Na+ uptake, survival and K+ uptake on SES scores in an F2:3 population of a cross between Capsule (salt tolerant) and BR29 (salt sensitive) grown under salt-stress at seedling stage. (PDF 843 kb) [file 12284_2019_319_MOESM1_ESM.pdf]

Additional file 1: Table S1a: Correlation and path coefficients for direct and indirect effects of Na^+ concentration, K^+ concentration, survival, Na^+-K^+ ratio, and shoot biomass on SES scores in an $F_{2:3}$ population of a cross between Capsule (salt tolerant) and BR29 (salt sensitive) grown under salt-stress at seedling stage.

Predictor variables	Correlation	Direct effects (D)	Indirect effects (I)						Total effects (D+I)
			Na^+ concentration	Survival	K^+ concentration	Na^+-K^+ ratio	Biomass (Shoot)	Total (I)	
Na^+ concentration	0.692	0.116		0.261	0.048	-0.004	0.270	0.575	0.691
Survival	-0.795	-0.368	0.083		-0.052	0.0036	-0.296	-0.427	-0.794
K^+ concentration	-0.184	-0.122	-0.112	0.072		-0.151	0.13	-0.061	-0.183
Na^+-K^+ ratio	0.440	0.0085	0.051	0.152	0.033		0.191	0.427	0.436
Biomass (Shoot)	-0.815	-0.427	-0.074	-0.255	-0.063	0.004		-0.388	-0.815

Residual effects= 0.50

Additional file 1: Table S 1b: Correlation and path coefficients for direct and indirect effects of Na^+ concentration, survival and K^+ concentration on SES scores in an $F_{2:3}$ population of a cross between Capsule (salt tolerant) and BR29 (salt sensitive) grown under salt-stress at seedling stage.

Predictor variables	Correlation	Direct effects (D)	Indirect effects (I)				Total effects (D+I)
			Na^+ concentration	Survival	K^+ concentration	Total (I)	
Na^+ concentration	0.692	0.215		0.387	0.089	0.477	0.692
Survival	-0.794	-0.547	-0.153		-0.095	-0.248	-0.795
K^+ concentration	-0.184	-0.139	-0.125	0.081		-0.044	-0.183

Residual effect= 0.61
