# Supplementary material for: Assessing trait contribution and mapping novel QTL for salinity tolerance using the Bangladeshi rice landrace Capsule
Source: Rice (N Y). 2019 Aug 13;12:63. doi: 10.1186/s12284-019-0319-5 (PMC6692794; doi:10.1186/s12284-019-0319-5)
Supplement: Supplementary file 2 — Table S2. χ2-test statistics testing of markers showing Mendelian inheritance for goodness of fit. (PDF 941 kb) [file 12284_2019_319_MOESM2_ESM.pdf]

Additional File 2: Table S2: χ2-test statistics testing of markers showing Mendelian inheritance for goodness of fit

Marker	Missing	Observed Frequency			Expected Frequency (1:2:1)			(O-E)^2/E			Total χ2 value
		BR29 (P1)	Hetero	Capsule (P2)	BR29 (P1)	Hetero	Capsule (P2)	BR29 (P1)	Hetero	Capsule (P2)	
**RM3252**	0	17	59	18	23.5	47	23.5	1.798	3.064	1.287	**6.149**
**RM428A**	1	21	48	24	23.5	47	23.5	0.266	0.021	0.011	**0.298**
**RM490**	0	18	46	30	23.5	47	23.5	1.287	0.021	1.798	**3.106**
**RM575**	9	16	42	27	23.5	47	23.5	2.394	0.532	0.521	**3.447**
**AP3206f**	0	23	42	29	23.5	47	23.5	0.011	0.532	1.287	**1.830**
**RM3412b**	0	22	48	24	23.5	47	23.5	0.096	0.021	0.011	**0.128**
**RM10793**	0	24	45	25	23.5	47	23.5	0.011	0.085	0.096	**0.191**
**RM562**	0	23	46	25	23.5	47	23.5	0.011	0.021	0.096	**0.128**
**RM449**	0	22	45	27	23.5	47	23.5	0.096	0.085	0.521	**0.702**
**RM11125**	5	19	41	29	23.5	47	23.5	0.862	0.766	1.287	**2.915**
**RM9**	0	10	51	33	23.5	47	23.5	7.755	0.340	3.840	**11.936**
**RM246**	2	22	51	19	23.5	47	23.5	0.096	0.340	0.862	**1.298**
**RM11570**	12	24	36	22	23.5	47	23.5	0.011	2.574	0.096	**2.681**
**RM472**	5	22	44	23	23.5	47	23.5	0.096	0.191	0.011	**0.298**
**RM12208**	1	23	51	19	23.5	47	23.5	0.011	0.340	0.862	**1.213**
**RM14**	0	18	50	26	23.5	47	23.5	1.287	0.191	0.266	**1.745**
**RM154**	0	20	59	15	23.5	47	23.5	0.521	3.064	3.074	**6.660**
**RM279**	0	30	46	18	23.5	47	23.5	1.798	0.021	1.287	**3.106**
**RM300**	0	26	46	22	23.5	47	23.5	0.266	0.021	0.096	**0.383**
**RM13197**	2	34	42	16	23.5	47	23.5	4.691	0.532	2.394	**7.617**
**RM2634**	2	22	48	22	23.5	47	23.5	0.096	0.021	0.096	**0.213**
**RM13628**	0	25	50	19	23.5	47	23.5	0.096	0.191	0.862	**1.149**
**RM525**	0	29	47	18	23.5	47	23.5	1.287	0.000	1.287	**2.574**
**RM208**	2	26	39	27	23.5	47	23.5	0.266	1.362	0.521	**2.149**
**RM22**	0	30	47	17	23.5	47	23.5	1.798	0.000	1.798	**3.596**

**RM14795**	6	19	48	21	23.5	47	23.5	0.862	0.021	0.266	**1.149**
**RM5928**	1	21	55	17	23.5	47	23.5	0.266	1.362	1.798	**3.426**
**RM3291**	1	24	48	21	23.5	47	23.5	0.011	0.021	0.266	**0.298**
**S03076B**	0	24	43	27	23.5	47	23.5	0.011	0.340	0.521	**0.872**
**RM251**	3	22	47	22	23.5	47	23.5	0.096	0.000	0.096	**0.191**
**RM4730**	7	16	53	18	23.5	47	23.5	2.394	0.766	1.287	**4.447**
**RM5626**	0	22	49	23	23.5	47	23.5	0.096	0.085	0.011	**0.191**
**RM6329**	1	25	50	18	23.5	47	23.5	0.096	0.191	1.287	**1.574**
**R3M53**	0	26	54	14	23.5	47	23.5	0.266	1.043	3.840	**5.149**
**RM148**	1	19	37	37	23.5	47	23.5	0.862	2.128	7.755	**10.745**
**RM16236**	5	30	41	18	23.5	47	23.5	1.798	0.766	1.287	**3.851**
**RM335**	0	25	49	20	23.5	47	23.5	0.096	0.085	0.521	**0.702**
**RM518**	1	22	49	22	23.5	47	23.5	0.096	0.085	0.096	**0.277**
**RM261**	10	14	34	36	23.5	47	23.5	3.840	3.596	6.649	**14.085**
**RM16686**	5	21	46	22	23.5	47	23.5	0.266	0.021	0.096	**0.383**
**S04065**	0	24	49	21	23.5	47	23.5	0.011	0.085	0.266	**0.362**
**RM16852**	14	16	43	21	23.5	47	23.5	2.394	0.340	0.266	**3.000**
**RM252A**	5	22	56	11	23.5	47	23.5	0.096	1.723	6.649	**8.468**
**RM3839**	0	18	52	24	23.5	47	23.5	1.287	0.532	0.011	**1.830**
**RM26212**	2	22	38	32	23.5	47	23.5	0.096	1.723	3.074	**4.894**
**RM280**	9	19	39	27	23.5	47	23.5	0.862	1.362	0.521	**2.745**
**RM17954**	1	14	57	22	23.5	47	23.5	3.840	2.128	0.096	**6.064**
**RM169**	0	24	41	29	23.5	47	23.5	0.011	0.766	1.287	**2.064**
**RM249**	0	26	41	27	23.5	47	23.5	0.266	0.766	0.521	**1.553**
**R5M20**	0	25	43	26	23.5	47	23.5	0.096	0.340	0.266	**0.702**
**RM163**	0	23	44	27	23.5	47	23.5	0.011	0.191	0.521	**0.723**
**RM3870**	0	21	52	21	23.5	47	23.5	0.266	0.532	0.266	**1.064**
**RM3809**	8	21	45	20	23.5	47	23.5	0.266	0.085	0.521	**0.872**
**RM19199**	10	33	29	22	23.5	47	23.5	3.840	6.894	0.096	**10.830**

**RM19238**	0	11	50	33	23.5	47	23.5	6.649	0.191	3.840	**10.681**
**RM402**	0	32	46	16	23.5	47	23.5	3.074	0.021	2.394	**5.489**
**R6M14**	1	35	39	19	23.5	47	23.5	5.628	1.362	0.862	**7.851**
**RM19840**	0	33	46	15	23.5	47	23.5	3.840	0.021	3.074	**6.936**
**RM3628**	0	30	43	21	23.5	47	23.5	1.798	0.340	0.266	**2.404**
**RM3138**	1	26	45	22	23.5	47	23.5	0.266	0.085	0.096	**0.447**
**RM20783**	9	17	44	24	23.5	47	23.5	1.798	0.191	0.011	**2.000**
**RM180**	1	27	46	20	23.5	47	23.5	0.521	0.021	0.521	**1.064**
**RM501A**	5	21	49	19	23.5	47	23.5	0.266	0.085	0.862	**1.213**
**RM336**	3	24	54	13	23.5	47	23.5	0.011	1.043	4.691	**5.745**
**RM3753**	0	25	57	12	23.5	47	23.5	0.096	2.128	5.628	**7.851**
**RM248**	0	38	44	12	23.5	47	23.5	8.947	0.191	5.628	**14.766**
**RM428B**	1	21	48	24	23.5	47	23.5	0.266	0.021	0.011	**0.298**
**RM152**	0	17	55	22	23.5	47	23.5	1.798	1.362	0.096	**3.255**
**RM547**	0	19	49	26	23.5	47	23.5	0.862	0.085	0.266	**1.213**
**RM22825**	8	20	41	25	23.5	47	23.5	0.521	0.766	0.096	**1.383**
**RM331**	0	16	53	25	23.5	47	23.5	2.394	0.766	0.096	**3.255**
**RM483**	10	23	44	17	23.5	47	23.5	0.011	0.191	1.798	**2.000**
**RM223**	0	15	57	22	23.5	47	23.5	3.074	2.128	0.096	**5.298**
**RM210**	10	17	51	18	23.5	47	23.5	1.798	0.340	1.287	**3.426**
**RM3571**	0	19	51	24	23.5	47	23.5	0.862	0.340	0.011	**1.213**
**RM3120**	4	30	37	23	23.5	47	23.5	1.798	2.128	0.011	**3.936**
**RM296**	0	14	63	17	23.5	47	23.5	3.840	5.447	1.798	**11.085**
**R9M10**	1	19	50	24	23.5	47	23.5	0.862	0.191	0.011	**1.064**
**RM6051**	0	18	57	19	23.5	47	23.5	1.287	2.128	0.862	**4.277**
**R9M30**	1	20	59	14	23.5	47	23.5	0.521	3.064	3.840	**7.426**
**RM242**	1	21	60	12	23.5	47	23.5	0.266	3.596	5.628	**9.489**
**RM24804**	10	19	44	21	23.5	47	23.5	0.862	0.191	0.266	**1.319**
**RM222**	0	28	42	24	23.5	47	23.5	0.862	0.532	0.011	**1.404**

**RM501C**	5	21	49	19	23.5	47	23.5	0.266	0.085	0.862	**1.213**
**R10M17**	0	28	43	23	23.5	47	23.5	0.862	0.340	0.011	**1.213**
**RM5806**	2	21	54	17	23.5	47	23.5	0.266	1.043	1.798	**3.106**
**RM304**	2	14	59	19	23.5	47	23.5	3.840	3.064	0.862	**7.766**
**RM496**	0	21	55	18	23.5	47	23.5	0.266	1.362	1.287	**2.915**
**RM25972**	5	21	50	18	23.5	47	23.5	0.266	0.191	1.287	**1.745**
**RM26212A**	2	22	38	32	23.5	47	23.5	0.096	1.723	3.074	**4.894**
**RM26237**	3	20	47	24	23.5	47	23.5	0.521	0.000	0.011	**0.532**
**RM3137**	0	21	45	28	23.5	47	23.5	0.266	0.085	0.862	**1.213**
**RM26652**	0	18	50	26	23.5	47	23.5	1.287	0.191	0.266	**1.745**
**RM21**	0	26	43	25	23.5	47	23.5	0.266	0.340	0.096	**0.702**
**RM6094**	0	21	51	22	23.5	47	23.5	0.266	0.340	0.096	**0.702**
**RM224**	0	18	52	24	23.5	47	23.5	1.287	0.532	0.011	**1.830**
**RM27421**	7	23	45	19	23.5	47	23.5	0.011	0.085	0.862	**0.957**
**RM27615**	4	20	49	21	23.5	47	23.5	0.521	0.085	0.266	**0.872**
**RM252B**	5	22	56	11	23.5	47	23.5	0.096	1.723	6.649	**8.468**
**RM27877**	0	21	60	13	23.5	47	23.5	0.266	3.596	4.691	**8.553**
**RM7102**	0	21	60	13	23.5	47	23.5	0.266	3.596	4.691	**8.553**
**S12055**	0	21	60	13	23.5	47	23.5	0.266	3.596	4.691	**8.553**
**R12M27**	0	15	60	19	23.5	47	23.5	3.074	3.596	0.862	**7.532**
**RM28466**	7	22	43	22	23.5	47	23.5	0.096	0.340	0.096	**0.532**
**RM17**	0	26	41	27	23.5	47	23.5	0.266	0.766	0.521	**1.553**

Tabulated χ^2 value = 5.99 and 9.21 at *P*<0.05 level and *P*<0.01 level, respectively

Out of 105 markers, 82 non-distorted markers
