# Supplementary material for: Assessing trait contribution and mapping novel QTL for salinity tolerance using the Bangladeshi rice landrace Capsule
Source: Rice (N Y). 2019 Aug 13;12:63. doi: 10.1186/s12284-019-0319-5 (PMC6692794; doi:10.1186/s12284-019-0319-5)
Supplement: Supplementary file 3 — Table S3. Segregation of SSR and InDel markers that were not associated with QTL for salinity tolerance in selected salt-tolerant and salt-sensitive classes in an F2 population of a cross between Capsule (salt tolerant) and BR29 (salt sensitive). (PDF 848 kb) [file 12284_2019_319_MOESM3_ESM.pdf]

Additional file 3: Table S3: Segregation of SSR and *InDel* markers that were not associated with QTLs for salinity tolerance in selected salt-tolerant and salt-sensitive classes in an F_2 population of a cross between Capsule (salt tolerant) and BR29 (salt sensitive).

Genetic markers	Tolerant class				Sensitive class				Difference		Confidence interval > 95% *i.e. (Pr-Ps)*$\geq 2\sigma_p$
	E/E	E/Cap	Cap	Pr^b	E/E	E/Cap	Cap	Ps^b	*Pr-Ps*	Standard error (σp)	
**Chromosome 1**											
RM3252	7	28	12	0.298	10	31	6	0.330	-0.032	0.0676	0.135
RM428A	10	22	15	0.234	12	26	9	0.277	-0.043	0.0635	0.127
RM490	6	22	19	0.234	12	24	11	0.255	-0.021	0.0627	0.125
RM575	6	19	22	0.202	20	18	9	0.191	0.011	0.0580	0.116
AP3206f	8	22	17	0.234	15	20	12	0.213	0.021	0.0607	0.121
RM3412b	7	26	14	0.277	15	20	12	0.213	0.064	0.0625	0.125
RM10793	10	24	13	0.255	14	21	12	0.223	0.032	0.0622	0.124
RM562	9	23	15	0.245	14	23	10	0.245	0.000	0.0627	0.125
RM449	13	22	12	0.234	9	23	15	0.245	-0.011	0.0622	0.124
RM11125	13	16	18	0.170	10	25	12	0.266	-0.096	0.0598	0.120
RM9	5	25	17	0.266	5	26	16	0.277	-0.011	0.0648	0.130
RM246	10	28	9	0.298	13	23	11	0.245	0.053	0.0647	0.129
RM11570	13	20	14	0.213	19	16	12	0.170	0.043	0.0573	0.115
**Chromosome 2**											
RM154	12	32	3	0.340	8	27	12	0.287	0.053	0.0676	0.135
RM279	13	23	11	0.245	17	23	7	0.245	0.000	0.0627	0.125
RM300	11	20	16	0.213	15	26	6	0.277	-0.064	0.0625	0.125
RM13197	28	8	11	0.085	33	8	6	0.085	0.000	0.0407	0.081
RM2634	10	23	14	0.245	12	25	10	0.266	-0.021	0.0636	0.127
RM13628	12	26	9	0.277	13	24	10	0.255	0.021	0.0644	0.129
**Chromosome 3**											
RM22	14	21	12	0.223	16	26	5	0.277	-0.053	0.0630	0.126
RM14795	6	24	17	0.255	13	24	10	0.255	0.000	0.0636	0.127
RM5928	6	31	10	0.330	15	24	8	0.255	0.074	0.0661	0.132
RM3291	15	20	12	0.213	9	28	10	0.298	-0.085	0.0633	0.127
S03076B	10	23	14	0.245	14	20	13	0.213	0.032	0.0612	0.122

Genetic markers	Tolerant class				Sensitive class				Difference		Confidence interval > 95% *i.e.* $(Pr\text{-}Ps)\geq 2\sigma_p$
	E/E	E/Cap	Cap	Pr^b	E/E	E/Cap	Cap	Ps^b	*Pr-Ps*	Standard error (σp)	
RM251	9	22	16	0.234	13	25	9	0.266	-0.032	0.0631	0.126
RM148	2	23	22	0.245	7	14	26	0.149	0.096	0.0576	0.115
RM16236	18	22	7	0.234	23	19	5	0.202	0.032	0.0602	0.120
**Chromosome 4**											
RM335	14	27	6	0.287	11	22	14	0.234	0.053	0.0639	0.128
RM518	12	27	8	0.287	11	22	14	0.234	0.053	0.0639	0.128
RM261	10	14	23	0.149	22	7	18	0.074	0.074	0.0456	0.091
RM16686	12	23	12	0.245	12	23	12	0.245	0.000	0.0627	0.125
S04065	10	24	13	0.255	14	25	8	0.266	-0.011	0.0640	0.128
RM16852	8	22	17	0.234	14	21	12	0.223	0.011	0.0613	0.123
RM252A	20	25	2	0.266	7	31	9	0.330	-0.064	0.0665	0.133
RM3839	6	29	12	0.309	12	23	12	0.245	0.064	0.0651	0.130
RM26212	9	22	16	0.234	13	16	18	0.170	0.064	0.0584	0.117
RM280	11	15	21	0.160	13	24	10	0.255	-0.096	0.0587	0.117
**Chromosome 5**											
RM17954	9	29	9	0.309	5	28	14	0.298	0.011	0.0670	0.134
RM169	8	19	20	0.202	16	22	9	0.234	-0.032	0.0602	0.120
RM249	9	19	19	0.202	17	22	8	0.234	-0.032	0.0602	0.120
R5M20	8	22	17	0.234	17	21	9	0.223	0.011	0.0613	0.123
RM163	9	19	19	0.202	14	25	8	0.266	-0.064	0.0616	0.123
**Chromosome 6**											
RM19238	8	20	19	0.213	3	30	14	0.319	-0.106	0.0640	0.128
RM402	16	26	5	0.277	16	20	11	0.213	0.064	0.0625	0.125
R6M14	17	23	7	0.245	18	16	13	0.170	0.074	0.0589	0.118
RM19840	17	21	9	0.223	16	25	6	0.266	-0.043	0.0626	0.125
RM3628	16	21	10	0.223	14	22	11	0.234	-0.011	0.0613	0.123
RM3138	13	18	16	0.191	13	27	7	0.287	-0.096	0.0618	0.124
**Chromosome 7**					47						
RM20783	6	18	23	0.191	20	16	11	0.170	0.021	0.0561	0.112
RM180	14	24	9	0.255	14	22	11	0.234	0.021	0.0627	0.125
RM501A	6	26	15	0.277	16	23	8	0.245	0.032	0.0640	0.128

Genetic markers	Tolerant class				Sensitive class				Difference		Confidence interval > 95% *i.e.* $(Pr\text{-}Ps)\geq 2\sigma_p$
	E/E	E/Cap	Cap	Pr^b	E/E	E/Cap	Cap	Ps^b	*Pr-Ps*	Standard error (σp)	
RM336	10	30	7	0.319	17	24	6	0.255	0.064	0.0658	0.132
RM3753	9	30	8	0.319	16	27	4	0.287	0.032	0.0670	0.134
RM248	17	21	9	0.223	21	23	3	0.245	-0.021	0.0617	0.123
RM428B	10	22	15	0.234	12	26	9	0.277	-0.043	0.0635	0.127
**Chromosome 8**											
RM152	9	29	9	0.309	8	26	13	0.277	0.032	0.0663	0.133
RM547	13	23	11	0.245	6	26	15	0.277	-0.032	0.0640	0.128
RM22825	12	20	15	0.213	13	21	13	0.223	-0.011	0.0602	0.120
RM331	11	26	10	0.277	5	27	15	0.287	-0.011	0.0656	0.131
RM483	12	26	9	0.277	19	18	10	0.191	0.085	0.0614	0.123
RM223	10	28	9	0.298	5	29	13	0.309	-0.011	0.0670	0.134
RM210	8	26	13	0.277	21	15	11	0.160	0.117	0.0596	0.119
RM3571	6	26	15	0.277	13	25	9	0.266	0.011	0.0648	0.130
RM3120	14	20	13	0.213	19	17	11	0.181	0.032	0.0579	0.116
**Chromosome 9**											
RM296	6	31	10	0.330	8	32	7	0.340	-0.011	0.0688	0.138
R9M10	10	33	4	0.351	10	24	13	0.255	0.096	0.0667	0.133
RM6051	9	26	12	0.277	9	31	7	0.330	-0.053	0.0669	0.134
R9M30	7	33	7	0.351	14	26	7	0.277	0.074	0.0675	0.135
RM242	7	31	9	0.330	12	33	2	0.351	-0.021	0.0691	0.138
RM24804	9	21	17	0.223	16	23	8	0.245	-0.021	0.0617	0.123
**Chromosome 10**					47						
RM222	12	22	13	0.234	16	20	11	0.213	0.021	0.0607	0.121
RM501C	6	26	15	0.277	16	23	8	0.245	0.032	0.0640	0.128
R10M17	12	22	13	0.234	16	21	10	0.223	0.011	0.0613	0.123
RM5806	10	26	11	0.277	12	28	7	0.298	-0.021	0.0660	0.132
RM304	9	30	8	0.319	6	29	12	0.309	0.011	0.0677	0.135
RM496	7	32	8	0.340	14	23	10	0.245	0.096	0.0660	0.132
**Chromosome 11**											
RM25972	9	27	11	0.287	14	23	10	0.245	0.043	0.0644	0.129
RM26212A	9	22	16	0.234	13	16	18	0.170	0.064	0.0584	0.117

Genetic markers	Tolerant class				Sensitive class				Difference		Confidence interval > 95% *i.e.* $(Pr\text{-}Ps)\geq 2\sigma_p$
	E/E	E/Cap	Cap	*Pr*[b]	E/E	E/Cap	Cap	*Ps*[b]	*Pr-Ps*	Standard error (σp)	
RM26237	10	25	12	0.266	12	22	13	0.234	0.032	0.0631	0.126
RM3137	10	23	14	0.245	11	22	14	0.234	0.011	0.0622	0.124
RM26652	8	27	12	0.287	10	23	14	0.245	0.043	0.0644	0.129
RM21	12	22	13	0.234	14	21	12	0.223	0.011	0.0613	0.123
RM6094	9	26	12	0.277	12	25	10	0.266	0.011	0.0648	0.130
RM224	8	27	12	0.287	10	25	12	0.266	0.021	0.0652	0.130
**Chromosome 12**											
RM27421	7	24	16	0.255	26	14	7	0.149	0.106	0.0581	0.116
RM27615	9	22	16	0.234	12	27	8	0.287	-0.053	0.0639	0.128
RM27877	6	32	9	0.340	15	28	4	0.298	0.043	0.0679	0.136
RM7102	6	32	9	0.340	15	28	4	0.298	0.043	0.0679	0.136
S12055	6	31	10	0.330	15	29	3	0.309	0.021	0.0680	0.136
R12M27	10	33	4	0.351	5	27	15	0.287	0.064	0.0678	0.136
RM28466	8	22	17	0.234	18	21	8	0.223	0.011	0.0613	0.123
RM17	12	22	13	0.234	14	19	14	0.202	0.032	0.0602	0.120

[a]E/E = homozygous for BR29 alleles; E/Cap = heterozygous
[b]Capsule allele frequency among the tolerant *(Pr)* and sensitive progeny *(Ps)*
