# Supplementary material for: Assessing trait contribution and mapping novel QTL for salinity tolerance using the Bangladeshi rice landrace Capsule
Source: Rice (N Y). 2019 Aug 13;12:63. doi: 10.1186/s12284-019-0319-5 (PMC6692794; doi:10.1186/s12284-019-0319-5)
Supplement: Supplementary file 4 — Table S4. Significant digenic/ epistatic interactions (P ≤ 1.0 × 10− 5) and LOD values for agronomic and physiological traits in an F2:3 population from a cross between Capsule/BR29. (PDF 843 kb) [file 12284_2019_319_MOESM4_ESM.pdf]

Additional file 4: Table S4: Significant digenic/ epistatic interactions ($P \leq 1.0 \times 10^{-5}$) and LOD values for agronomic and physiological traits in an $F_{2:3}$ population from a cross between Capsule/BR29.

Trait	Chr-A	Locus-A	Chr-B	Locus-B	Total LOD for association (LODt)	Interaction LOD (IX)	LOD for Locus-A main effect (Main-A)	LOD for Locus-B main effect (Main-B)	Type of interaction
Survival	3	RM5928	3	RM6329	8.15	3.07	1.07	3.65	Between QTL and Background
	3	RM14795	3	RM6329	10.17	5.35	0.24	3.70	Between QTL and Background
	3	RM251	3	RM6329	8.15	3.59	0.37	3.70	Between QTL and Background
SES	3	RM5928	3	RM6329	10.13	3.24	1.04	5.18	Between QTL and Background
	3	RM14795	3	RM6329	10.76	4.43	0.39	5.26	Between QTL and Background
	3	RM251	3	RM6329	9.78	3.13	0.80	5.26	Between QTL and Background
