# Supplementary material for: Assessing trait contribution and mapping novel QTL for salinity tolerance using the Bangladeshi rice landrace Capsule
Source: Rice (N Y). 2019 Aug 13;12:63. doi: 10.1186/s12284-019-0319-5 (PMC6692794; doi:10.1186/s12284-019-0319-5)
Supplement: Supplementary file 5 — Figure S1. Chromosomal constitution of five salt-tolerant and five salt-sensitive F2 individuals for chromosome 1 and 3. The sky blue area indicates heterozygous genotypes at the marker loci, the green area indicates homozygous donor parent genotypes and the red color area indicate sensitive parent BR29 alleles. The names of markers are listed at the top of the figure. It is apparent that the salt tolerant individuals had the favorable QTL alleles (homozygous or heterozygote alleles from Capsule) and the salt sensitive individuals had opposite QTL alleles from BR29 at the QTL loci (RM12208 and RM6329). (PDF 843 kb) [file 12284_2019_319_MOESM5_ESM.pdf]

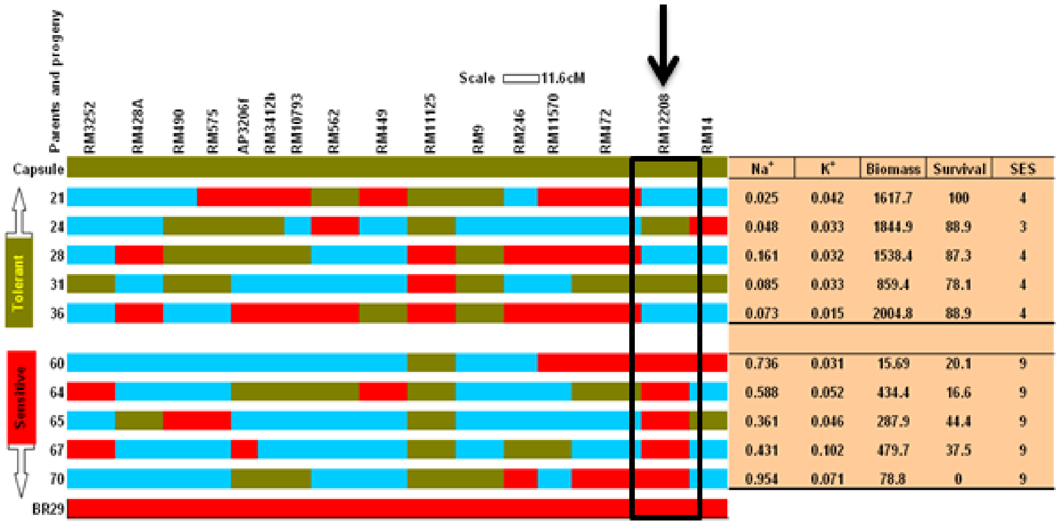


Chromosome 1

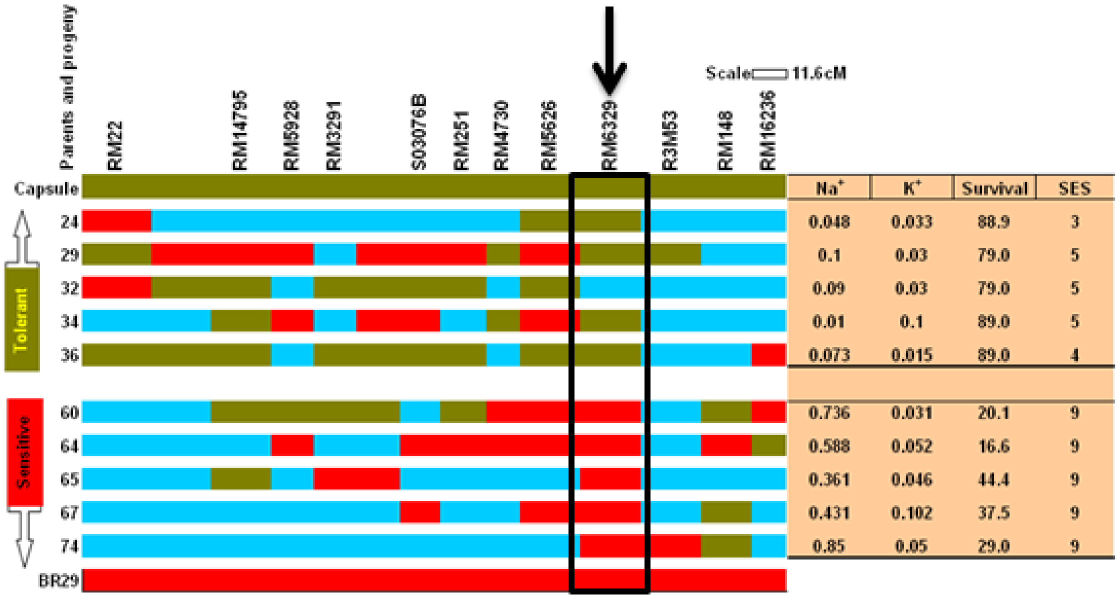


Chromosome 3

Additional file 5: Figure S1: Chromosomal constitution of five salt-tolerant and five salt-sensitive $F_2$ individuals for chromosome 1 and 3. The sky blue area indicates heterozygous genotypes at the marker loci, the green area indicates homozygous donor parent genotypes and the red color area indicate sensitive parent BR29 alleles. The names of markers are listed at the top of the figure. It is apparent that the salt tolerant individuals had the favorable QTL alleles (homozygous or heterozygote alleles from Capsule) and the salt sensitive individuals had opposite QTL alleles from BR29 at the QTL loci (RM12208 and RM6329).
