# Supplementary material for: Assessing trait contribution and mapping novel QTL for salinity tolerance using the Bangladeshi rice landrace Capsule
Source: Rice (N Y). 2019 Aug 13;12:63. doi: 10.1186/s12284-019-0319-5 (PMC6692794; doi:10.1186/s12284-019-0319-5)
Supplement: Supplementary file 6 — Table S5. Comparisons of the QTL regions identified in this study for salinity tolerance at seedling stage, with previously mapped QTL from different populations and for different growth stages. (PDF 845 kb) [file 12284_2019_319_MOESM6_ESM.pdf]

Additional file 6: Table S5: Comparisons of the QTL regions identified in this study for salinity tolerance at seedling stage, with previously mapped QTLs from different populations and for different growth stages

Gene/QTL name	Chr	Growth stage	Mapping population	Evaluation index of salinity tolerance	Interval/peak marker	Interval distance (cM or Mb)	PVE (%)	References*	Remarks (Novel /Same QTLs/Absent)
Q_{Na}	1	Seedling	RIL	High Na uptake				Flowers et al. 2000	Absent
Trait-based QTL	1	Seedling	RIL	Na^+ uptake	E12M55-3	74 cM	8.9	Koyama et al. 2001	,,
Trait-based QTL	1	Seedling	RIL	K^+ concentration	E12M37-1	56 cM	10.6	*qK1.1* (162.9 cM)- This study	Novel
Trait-based QTL	1	Seedling	RIL	Na: K ratio	E12M57-1	74 cM	9.1		Absent
Saltol	1	Seedling	RIL	Na^+ uptake	RM140 - C1733S	between 51.6 and 65.9cM / 13.87 Mb	39.2	Bonilla et al. 2002 *qNa1.1* (162.9 cM)- This study	Novel
Saltol	1	Seedling	RIL	K^+ uptake	RM140 - C1733S	between 51.6 and 65.9cM / 13.87 Mb	43.9	*qK1.1* (162.9 cM)	,,
Saltol	1	Seedling	RIL	Na/K ratio	RM140 - C1733S	between 51.6 and 65.9cM / 13.87 Mb	43.2	*qNaK-R1.1* (162.9 cM)	,,
qSDS-1	1	Seedling	$F_{2:3}$	Seedling survival	C813 - C86	-	18.0	Lin et al. 2004 *qSur1.1* (162.9 cM)- This study	Novel
qSKC-1	1	Seedling	$F_{2:3}$	Shoot K^+ concentration	C1211 - S2139	-	40.1		Absent
qRNTQ-1	1	Seedling	$F_{2:3}$	Root Na^+ total quantity	C813 - C86	-	12.4		,,
OsHKT1;5 (SKC1)	1	Seedling	Advanced backcross	Shoot K^+ concentration	K159 - K061	11.46 Mb	-	Ren et al. 2005	Absent
qST1	1	Seedling	RIL		Est1-2 & RZ569A	40 cM	27.8	Lee et al. 2006	,,
qSES1	1	Seedling	F2	SES	RM8094-RM582	-	19.6	Bimpong et al. 2014 *qSES3.1* (111.0	Novel

								cM)	
qSL1.1	1	Seedling	RIL	Shoot length	id1023892–id1024836	162.6 cM	20.6	Bizimana et al. 2017 *qSL1.2* (162.9 cM)- This study	Same
qSL1.2	1	Seedling	RIL	Shoot length	id1024972–id1025983	168.6 cM	11.8		Absent
qSIS1.39	1	Seedling	IL	Salt injury score (SIS)	RM3810	39.5 cM	6.4	De Leon et al. 2017 *qSES3.1* (111.0 cM)- This study	Novel
qSHL1.39	1	Seedling	IL	Shoot length	RM3810	39.5 cM	8.2	*qSL1.1* (25.9 cM)- This study	Same
qSHL1.41	1	Seedling	IL	Shoot length	RM5362	41.1 cM	6.8		-
qSHL2.3	2	Seedling	IL	Shoot length	RM211	3.0 cM	6.6		Absent
qSES2.1	2	Seedling	RIL	SES	id2004774–id2007526	64.8 cM	11.1	Bizimana et al. 2017	,,
qSIS2.3	2	Seedling	IL	Salt injury score (SIS)	RM211	3.0 cM	6.5	*qSES5.2* (88.0 cM)- This study	,,
qSHL2.3	2	Seedling	IL	Shoot length	RM211	3.0 cM	6.6		Absent
qST3	3	Seedling	RIL		RG179 - RZ596	138 cM	9.2	Lee et al. 2006 *qNa3.3* (111.0 cM); *qK3.2* (111.0 cM); *qNaK-R3.3* (109.0 cM)- This study	Novel
Q_{NaK}	4	Seedling	RIL	Na:K discrimination				Flowers et al. 2000 *qNaK-R2.2* (109.0 cM)-This study	,,
Trait-based QTL	4	Seedling	RIL	K^+ uptake	E12M65-1	10 cM	6.8	Koyama et al. 2001	Novel
Trait-based QTL	4	Seedling	RIL	K^+ concentration	E15M53-2	90 cM	8.8	,,	,,
	4	Seedling	RIL	Na^+ concentration	E12M73-1; E12M75-5; E15M50-5; E12M79-1	24 cM	6.7	*qNa2.2* (109.0 cM)- This study	,,

Trait-based QTL	4	Seedling	RIL	Na:K ratio	E12M65-1	14 cM	9.6	*qNaK-R2.2* (109.0 cM)-This study	,,
qRKC-4	4	Seedling	$F_{2:3}$	Root K^+ concentration	C891 - C513	-	21.6	Lin et al. 2004	,,
qSDM-5	5	Seedling	Double haploid lines	Seedling dry matter	RZ70-RZ225	-	17.9	Prasad et al. 2000<br>*qSL5.3; qNaK-R5.4* (88.0 cM)- This study	,,
qSHL-5	5	Seedling	F2:4	Shoot length	RM13-RM164	106.8 cM	19.5	Ghomi et al. 2013	Absent
qSHL5.04	5	Seedling	IL	Shoot length	RM17749	0.4 cM	8.5	De Leon et al. 2017	,,
Q_{K1}	6	Seedling	NIL	K^+ uptake				Flowers et al. 2000	,,
qSGEM-6	6	Germination	Double haploid lines	Seed germination	RZ398-RG213	-	16.3	Prasad et al. 2000	,,
Trait-based QTL	6	Seedling	RIL	Dry mass	E12M55-2	34 cM	9.7	Koyama et al. 2001	Novel
Trait-based QTL	6	Seedling	RIL	K^+ uptake	OSR19; E12M80-2	30 cM	7.6	,,	Absent
Trait-based QTL	6	Seedling	RIL	Na^+ concentration	E12M35-2	106 cM	6.4	,,	,,
qSDS-6	6	Seedling	$F_{2:3}$	Seedling survival	C214 - R2549	-	17.0	Lin et al. 2004<br>*qSur3.2* (111.0 cM)- This study	Novel
qSES6	6	Seedling	F2	SES	RM586-RM253	-	39.7	Bimpong et al. 2014<br>*qSES5.2* (88.0 cM)- This study	,,
qSHL-6	6	Seedling	F2:4	Shoot length	RM402-RM549	87.4 cM	14.6	Ghomi et al. 2013	,,
qSL6.1	6	Seedling	RIL	Shoot length	fd13–id6004343	18.5 cM	12.1	Bizimana et al. 2017<br>*qSL5.3* (88.0 cM)- This study	Novel
qSIS6.5	6	Seedling	IL	Salt injury score (SIS)	RM253	5.4 cM	7.0	De Leon et al. 2017	,,

qSHL6.5	6	Seedling	IL	Shoot length	RM253	5.4 cM	12.6	,,	Absent
qSDS-7	7	Seedling	$F_{2:3}$	Seedling survival	R2401 - L538T7	-	13.9	Lin et al. 2004	,,
qSNC-7	7	Seedling	$F_{2:3}$	Shoot Na^+ concentration	C1057 - R2401	-	48.5	,,	Absent
qSNTQ-7	7	Seedling	$F_{2:3}$	Shoot Na^+ total quantity	C1057 - R2401	-	16.1	,,	,,
qRKC-7	7	Seedling	$F_{2:3}$	Root K^+ concentration	C1057 - R2401	-	17.8	,,	Absent
qRKTQ-7	7	Seedling	$F_{2:3}$	Root K^+ total quantity	C1057 - R2401	-	17.3	,,	,,
qSIS7.12	7	Seedling	IL	Salt injury score (SIS)	RM214	12.8 cM	5.9	De Leon et al. 2017	,,
qSIS7.17	7	Seedling	IL	Salt injury score (SIS)	RM5793	17.5 cM	8.1	,,	,,
qSHL7.12	7	Seedling	IL	Shoot length	RM214	12.8 cM	6.7	,,	,,
Q_{K2}	9	Seedling	NIL	K^+ uptake				Flowers et al. 2000	Absent
Trait-based QTL	9	Seedling	RIL	K^+ uptake	E12M55-4	96 cM	19.6	Koyama et al. 2001	,,
qRNC-9	9	Seedling	$F_{2:3}$	Root Na^+ concentration	R1751 - R2638	-	16.7	Lin et al. 2004	,,
qSHL-9	9	Seedling	F2:4	Shoot length	E37-M60-13-E36-M60-1	136.1 cM	9.9	Ghomi et al. 2013	,,
qSES10	10	Seedling	F2	SES	RM228-RM333	-	30.7	Bimpong et al. 2014	Absent
qSHL-10	10	Seedling	F2:4	Shoot length	RM2863-E36-M61-13	111.4 cM	4.1	Ghomi et al. 2013	,,
qSES11	11	Seedling	F2	SES	RM536-RM287	-	37.2	Bimpong et al. 2014	,,
qSL12.1	12	Seedling	RIL	Shoot length	id12000252–id12001321	6.9 cM	9.8	*qSES12.3* (31.0 cM)- This study Bizimana et al. 2017 *qSur12.3* (67.1 cM)-This study	Novel
qSES12.1	12	Seedling	RIL	SES	id12000252-id12001321	6.9 cM	10.6	*qSES12.3* (31.0 cM) - This study	,,

Chr: Chromosome number; IL: Introgression line; PVE: Phenotypic variation explained; RIL: Recombinant inbred line; SES: Overall phenotypic performance; SL: Shoot length; Na: Na$^{+:}$ concentration; K$^{:}$ K$^{+:}$ concentration; NaK-R: Na-K ratio, Sur: Survival; * indicates embedded QTLs identified in this study
