# Supplementary material for: Assessing trait contribution and mapping novel QTL for salinity tolerance using the Bangladeshi rice landrace Capsule
Source: Rice (N Y). 2019 Aug 13;12:63. doi: 10.1186/s12284-019-0319-5 (PMC6692794; doi:10.1186/s12284-019-0319-5)
Supplement: Supplementary file 7 — Figure S2. Summary of three major loci identified in this study. A, B, and C refer to the Saltol region on the short arm of chromosome 1, which was also identified before (Bonilla et al. 2002; Lin et al. 2004; Ren et al. 2005), whereas D and E represent two novel genomic regions identified in this study on the long arms of chromosome 1 and 3, respectively. (PDF 843 kb) [file 12284_2019_319_MOESM7_ESM.pdf]

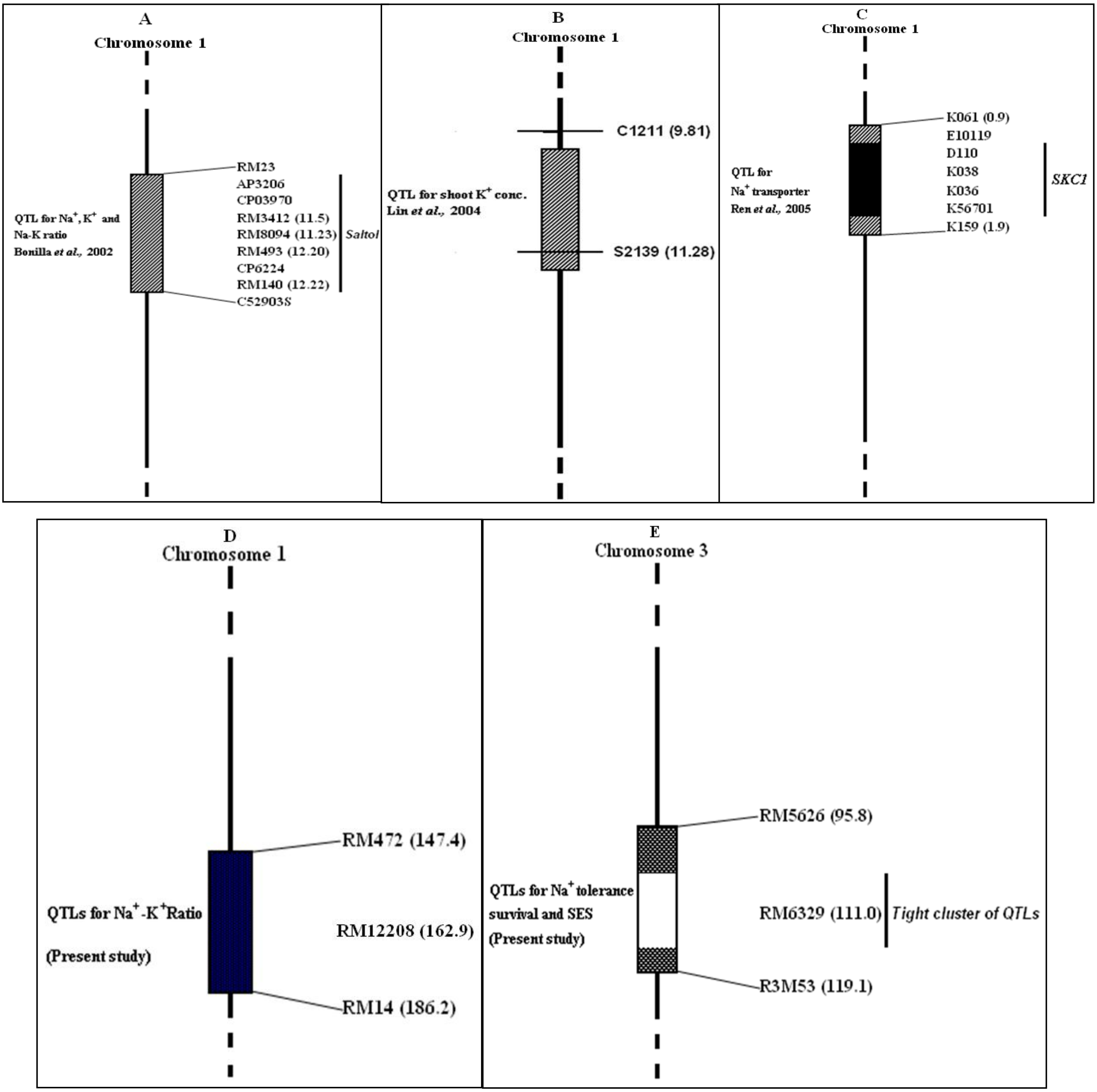

Additional file 7: Figure S2: Summary of three major loci identified in this study. A, B, and C refer to the *Saltol* region on the short arm of chromosome 1, which was also identified before (Bonilla et al. 2002; Lin et al. 2004; Ren et al. 2005), whereas D and E represent two novel genomic regions identified in this study on the long arms of chromosome 1 and 3, respectively.
